# PRR15 deficiency facilitates malignant progression by mediating PI3K/Akt signaling and predicts clinical prognosis in triple-negative rather than non-triple-negative breast cancer

**DOI:** 10.1038/s41419-023-05746-8

**Published:** 2023-04-18

**Authors:** Fengzhu Guo, Jialu Ma, Cong Li, Shuning Liu, Weizheng Wu, Chunxiao Li, Jiani Wang, Jinsong Wang, Zhijun Li, Jingtong Zhai, Fangzhou Sun, Yantong Zhou, Changyuan Guo, Haili Qian, Binghe Xu

**Affiliations:** 1 Department of Medical Oncology, National Cancer Center/National Clinical Research Center for Cancer/Cancer Hospital, Chinese Academy of Medical Sciences and Peking Union Medical College, Beijing, 100021 China; 2 State Key Laboratory of Molecular Oncology, National Cancer Center/National Clinical Research Center for Cancer/Cancer Hospital, Chinese Academy of Medical Sciences and Peking Union Medical College, Beijing, 100021 China; 3 Department of Urology, National Cancer Center/National Clinical Research Center for Cancer/Cancer Hospital, Chinese Academy of Medical Sciences and Peking Union Medical College, Beijing, 100021 China; 4 Graduate School, Hebei Medical University, Shijiazhuang, 050000 Hebei Province China; 5 Department of General Surgery, Affiliated Hospital of Zunyi Medical University, Zunyi, Guizhou 563000 China; 6 Department of Pathology, National Cancer Center/National Clinical Research Center for Cancer/Cancer Hospital, Chinese Academy of Medical Sciences and Peking Union Medical College, Beijing, 100021 China

**Keywords:** Breast cancer, Prognostic markers, Translational research, Metastasis, Cancer therapy

## Abstract

Triple-negative breast cancer (TNBC) is the most aggressive subtype of breast neoplasms with a higher risk of recurrence and metastasis than non-TNBC. Nevertheless, the factors responsible for the differences in the malignant behavior between TNBC and non-TNBC are not fully explored. Proline rich 15 (PRR15) is a protein involved in the progression of several tumor types, but its mechanisms are still controversial. Therefore, this study aimed to investigate the biological role and clinical applications of PRR15 on TNBC. PRR15 gene was differentially expressed between TNBC and non-TNBC patients, previously described as an oncogenic factor in breast cancer. However, our results showed a decreased expression of PRR15 that portended a favorable prognosis in TNBC rather than non-TNBC. PRR15 knockdown facilitated the proliferation, migration, and invasive ability of TNBC cells in vitro and in vivo, which was abolished by PRR15 restoration, without remarkable effects on non-TNBC. High-throughput drug sensitivity revealed that PI3K/Akt signaling was involved in the aggressive properties of PRR15 silencing, which was confirmed by the PI3K/Akt signaling activation in the tumors of PRR15^Low^ patients, and PI3K inhibitor reversed the metastatic capacity of TNBC in mice. The reduced PRR15 expression in TNBC patients was positively correlated with more aggressive clinicopathological characteristics, enhanced metastasis, and poor disease-free survival. Collectively, PRR15 down-regulation promotes malignant progression through the PI3K/Akt signaling in TNBC rather than in non-TNBC, affects the response of TNBC cells to antitumor agents, and is a promising indicator of disease outcomes in TNBC.

## Introduction

Triple-negative breast cancer (TNBC) is a subtype of breast cancer in which the expression of estrogen receptor (ER), progesterone receptor (PR), and human epidermal growth factor receptor 2 (HER2) is negative, representing approximately 15%–20% of breast neoplasms [1]. TNBC differs from other subsets of mammary neoplasms for its more malignant behavior as well as high risk of recurrence and death within the first five years after diagnosis [2]. Conventional chemotherapy still represents the mainstay of therapeutic strategies against TNBC due to the lack of effective receptor targets, making TNBC resistant to chemotherapeutic agents, thereby resulting in a dismal prognosis [3, 4]. Most of the mortality of TNBC patients is due to metastases, and the average survival of patients with advanced TNBC is two months, dramatically shorter than the period of survival reported in other subsets of breast cancer [5]. Therefore, in-depth research on the molecular mechanisms of breast cancer progression is of the utmost importance, as well as the identification of biomarkers capable of predicting the clinical outcomes, providing novel strategies for breast cancer treatment and patient management.

Proline rich 15 (PRR15) is a low molecular weight (approximately 13.7 kDa) and well-conserved protein initially isolated from the murine intestinal epithelium [6]. Glover and Seidel independently identified PRR15 using the mRNA differential display analysis in elongating bovine embryos. Its open reading frame encodes 126 amino acids, with a nuclear targeting sequence, four putative protein kinase C phosphorylation sites and two casein kinase II phosphorylation sites, as demonstrated by bioinformatics of the cDNA [7]. The research available on PRR15 is scarce, focusing on embryonic development, neurological diseases, and several tumor studies [8–15].

The role of PRR15 in tumor progression is still controversial. A study investigating the expression of *Prr15*/*PRR15* in gastrointestinal (GI) neoplasia indicated that *Prr15*/*PRR15* is differentially expressed in mouse GI tumors and human colorectal cancer (CRC) caused by different gene mutations, with high *Prr15*/*PRR15* levels in murine GI tumors resulting from mutations in the *Apc* gene, in conjunction with human CRCs. Since a large fraction of sporadic human CRCs carries *APC* mutations, these findings suggest that *Prr15/PRR15* expression may be directly or indirectly associated with *APC* deletion and Wnt signaling disruption [8]. Xing S et al. observed increased PRR15 in colon adenocarcinoma than in normal controls, and discovered that PRR15 is a crucial gene involved in the treatment of colon cancer with irinotecan and the Chinese herbal preparation PHY906 through the induction of cell apoptosis by the generation of reactive oxygen species, and inhibition of bacterial growth and inflammation [9]. In addition, in silico analysis of breast cancer revealed that both PRR15 mRNA and protein expression increases and the gene is hypomethylated in the tumor tissues compared with the healthy counterparts. PRR15 is inhibited in the signaling pathway networks involved in epithelial-mesenchymal transition (EMT) and activated in the ER pathway, exerting a pro-tumorigenic effect [10]. Instead, a study presenting opposite conclusions illustrated that the CpG of PRR15 is significantly methylated in bronchial washings from non-small cell lung cancer (NSCLC) patients, leading to its reduced expression. Smoking cessation decreases the DNA methylation of non-malignant bronchial epithelial cells in a gene-specific mode [11].

The existing findings on PRR15 and neoplasms remain poorly defined and underexplored, with basic studies mainly related to CRC and NSCLC and limited to bioinformatics in breast cancer. PRR15 was identified as a differentially expressed gene (DEG) between TNBC and non-TNBC. Therefore, the aim of the present study was to investigate the effect of PRR15 on TNBC and its downstream mechanisms. The effect of PRR15 dysregulation associated to the sensitivity of TNBC to specific drugs was also assessed, as well as the evaluation of the clinicopathological features correlated with PRR15 expression, and the prognostic value of PRR15 in patients with TNBC. Our results might reveal novel approaches in the improvement of individualized therapy for TNBC based on molecular typing.

## Materials and methods

### Cell culture and reagents

Non-cancerous mammary epithelium cell (MCF10A), breast cancer cells including luminal (MCF7, MDA-MB-361, T47D, and BT474), HER2amp (SKBR3), and triple-negative (MDA-MB-231, CAL51, BT20, and MDA-MB-468) subtypes, as well as human embryonic kidney 293T (HEK-293T) cells, were purchased from the American Type Culture Collection (Manassas, VA, USA). MDA-MB-231, CAL51, BT20, MDA-MB-468, MCF7, MDA-MB-361, and HEK-293T cells were cultured in Dulbecco’s Modified Eagle Medium (Hyclone, Logan, UT, USA) while T47D, BT474, and SKBR3 cells were grown in RPMI-1640 (Hyclone), both supplemented with 10% fetal bovine serum (FBS, Gibco, Grand Island, NY, USA) and 1% penicillin-streptomycin (Hyclone). MCF10A cells were maintained in mammary epithelial basal medium (Lonza, Walkersville, MD, USA) containing 100 ng/mL cholera toxin, 3 mg/mL bovine pituitary extract, 10 μg/mL hEGF, 0.5 mg/mL hydrocortisone, and 5 mg/mL insulin. All cells were incubated under a humidified atmosphere of 5% CO_2_ at 37 °C. Short tandem repeat profiling was performed by GENEWIZ (Suzhou, China), and mycoplasma tests were carried out using the MycoAlert Mycoplasma Detection Kit (Lonza).

The chemical reagents used in this study were purchased from the following providers: LY294002 from MedChemExpress (Shanghai, China, HY-10108), and the library containing 397 approved antineoplastics from TargetMol (Shanghai, China, L2110).

### Cell proliferation assay

The proliferation of cells was detected by the IncuCyte Live-Cell Analysis System (Essen BioScience, Ann Arbor, MI, USA). Briefly, cells were seeded in a 96-well plate at a density of 3 × 10^3^ cells per well. The plate was transferred into the IncuCyte platform after 24 h, and two sets of phase contrast images from different regions in each well were captured. Real-time proliferation was measured for 48 h or 72 h through the assessment of the percentage of cell confluence.

Cell viability was quantified by CCK8 assay. Cells were treated with the indicated agents and cultured in a 384-well plate for 72 h. CCK8 reagent (TargetMol) was added into each well and incubated for 1 h and the absorbance was measured at 450 nm using the microplate reader.

### Wound healing assay

The wound healing assay was carried out using the IncuCyte platform (Essen BioScience). Cells were seeded in a 96-well plate until they reached confluent monolayers, and homogeneous linear scratches were created by the IncuCyte WoundMaker. Then, the monolayers were rinsed with PBS to remove cell residues, and sequential images were automatically taken to monitor wound closure.

### Transwell migration and invasion assay

As regards the invasion assay, the 8 µm porous membrane (Corning, New York, NY, USA) of the transwell filter insert was coated with 0.5% Matrigel (Corning). Cells were seeded in the upper compartment containing serum-free medium at a density of 5 × 10^5^ cells/mL, while medium with 20% FBS was placed in the lower chamber as a chemoattractant. The non-invading or non-migrated cells adherent to the top of the membrane were removed by scrubbing after 24 h or 20 h of incubation. The invading or migrated cells adherent to the opposite side of the membrane were fixed with methanol for 10 min, stained with 0.5% crystal violet for 5 min, and five randomly selected fields were imaged under a light microscope.

### In vivo mouse experiments

#### Mice

The animal studies were performed in accordance with the protocol approved by the Ethical Committee for animal experiments of Cancer Hospital, Chinese Academy of Medical Science (CHCAMS). Four- to five-week-old female Balb/c-nude mice were purchased from HFK Bio-Technology (Beijing, China) and four-week-old female NOG mice were purchased from Charles River (Beijing, China). All mice were housed in pathogen-free, barrier-protected conditions at the CHCAMS animal facility, and were kept to acclimatize to the environment for one week prior to the experiments. Sample sizes for animal experiments were determined based upon pilot experiments. Mice were randomized into groups based on age and weight, and were not subjected to data blinding.

#### Xenograft

TNBC cells (MDA-MB-231 or CAL51, 3 × 10^6^) and non-TNBC cells (MCF7, 3 × 10^6^) with or without PRR15 knockdown were subcutaneously inoculated into the right flanks of 5-week-old Balb/c-nude mice and NOG mice, respectively. The tumor volume was measured by a caliper three times per week to assess the growth change. Mice were sacrificed at the end of the study or when the tumor grew enough to require euthanasia, and the tumors were extracted, weighed, and processed for further research. The volume was calculated as follows: 0.5 × length × width^2^.

#### Pulmonary metastatic model and treatment

PRR15-silenced MDA-MB-231 cells (5 × 10^5^) or controls were injected into the tail vein of 5-week-old immunodeficient mice. The mice were randomly divided into 2 groups 2 weeks after tail vein injection, and were treated with an intraperitoneal administration of the PI3K inhibitor LY294002 (75 mg/kg) or vehicle twice per week. All mice were euthanized after 2 weeks of consecutive treatment, and lungs were collected, weighed, and subjected to subsequent histopathological analysis. Metastatic nodules were estimated by the gross examination of freshly resected lungs and histological evaluation of hematoxylin and eosin (H&E)-stained sections.

### Constructs, transfection, and lentiviral infection

Stable knockdown of PRR15 was achieved in MDA-MB-231, CAL51, MCF7, and T47D cells by lentivirus-mediated RNA interference using validated shRNA sequences inserted in the lentivector pLKO.1. Randomized oligonucleotides were subcloned in the same vector and used as a control. Human DNA fragment encoding PRR15 was synthesized and incorporated into pCHD and pcDNA3.1 vectors to generate stable and transient overexpression plasmids, respectively, with the corresponding empty vectors used as controls. Details are available in Table S1.

As regards transient transfection, target cells were seeded in a 6-well plate and incubated overnight at 37 °C. Transfection mixes composed of 7.5 μL Lipofectamine 3000 (ThermoFisher, Waltham, MA, USA) in 125 μL Opti-MEM and 2.5 μg DNA together with 5 μL P3000 in 125 μL Opti-MEM (per well) were added to the wells according to the manufacturer’s instructions. As regards recombinant lentiviral particle production and infection, HEK-293T cells were co-transfected with knockdown or overexpression constructs and packaging plasmids (pMD2.G and psPAX) using Lipofectamine 3000 as described above. Virus-containing supernatants were collected after transfection, filtered, and supplemented with 5 μg/ml polybrene to infect target cells at 48 h and 72 h. After 24 h from the second infection, target cells were selected using 1.5 μg/mL puromycin dissolved in the medium for at least 7d.

### Reverse transcription quantitative polymerase chain reaction (RT-qPCR)

Total RNA was isolated from the suitably treated cells using Trizol (ThermoFisher) according to the provider’s protocol and quantified on the NanoDrop. cDNA was synthesized from 1 μg RNA using the high-capacity cDNA reverse transcription kit (ThermoFisher). The reaction was performed at 37 °C for 2 h and 85 °C for 5 min. RT-qPCR was performed using the Power SYBR Green master mix (ThermoFisher) under the following conditions: polymerase activation at 95 °C for 10 min, denaturation at 95 °C for 15 s, annealing and extension at 60 °C for 60 s for a total of 40 cycles. The primers specific for *PRR15*, *MKI67*, *PCNA*, *VIM*, *SNAI1*, *GAPDH*, and *ACTIN* are listed in Table S1.

### Immunoblotting

Cells were lysed using RIPA lysis buffer (Beyotime, Shanghai, China) containing protease and phosphatase inhibitors. Proteins were separated by SDS-PAGE gel and transferred by electrophoresis onto a polyvinylidene fluoride membrane. Subsequently, the membrane was blocked with 5% bovine serum albumin or skim milk in phosphate-buffered saline containing 0.05% Tween-20 followed by hybridization with appropriate primary antibodies at the dilutions recommended by the suppliers and incubated overnight at 4 °C. Next, the membrane was incubated with horseradish peroxidase (HRP)-conjugated secondary antibody, and the bands were visualized using enhanced chemiluminescence substrate (ThermoFisher, 32106). The primary antibodies were the following: PRR15 (RayBiotech, Norcross, GA, USA, 102-22108), PI3K (CST, Boston, MA, USA, 4292S), p-PI3K (Abcam, Cambridge, MA, USA, ab182651), Akt (CST, 4691S), p-Akt (CST, 4060S), mTOR (CST, 2972S), p-mTOR (CST, 2971S), N-cadherin (CST, 13116S), vimentin (Abclonal, Wuhan, China, A2584), Snail1(Invitrogen, Carlsbad, CA, USA, 14-9859-82), and β-actin (CST, 4967) used as a loading control. The intensity of the blots was quantified with software ImageJ and normalized to that of β-actin.

### Tissue sample collection, histology, and immunohistochemistry (IHC)

Two batches of patient samples were used in this study. One batch was represented by breast cancer tissue microarray purchased from Shanghai Outdo Biotech (Shanghai, China), and the other batch was represented by paraffin-embedded specimens of TNBC patients that were obtained from the Department of Pathology at CHCAMS after surgical resection.

Mouse tumor and lung tissues were embedded in paraffin after fixation in 10% formalin. Five-μm sections were stained with DAKO Envision Kit following dewaxing, hydration, antigen retrieval, along with peroxidase activity quenching. Slides were blocked to prevent nonspecific staining, followed by incubation with the primary antibody and HRP-conjugated secondary antibody, color development with diaminobenzidine, and counterstained with hematoxylin. The average intensity and the proportion of cells positive for each marker were scored by experienced pathologists to assess protein expression.

The protocol regarding the collected human samples was approved by the Ethics Committee of CHCAMS and written informed consent was obtained from each enrolled patient.

### RNA sequencing (RNA-seq)

RNA samples (three replicates per group) were isolated as described above and processed for mRNA library establishment and sequencing at BGI (Wuhan, China). The sequencing platform was MGISEQ-2000 and the paired-end reads were 100/150 bp. As regards the downstream analysis, raw sequencing data were filtered using SOAPnuke (v1.5.2), and then stored in FASTQ format as clean reads. Then, they were mapped to the reference genome using HISAT2 (v2.0.4), the clean files were aligned to the reference coding gene set using Bowtie2 (v2.2.5), and the expression of genes was acquired using RSEM (v1.2.12). Subsequently, DEGs were identified by DESeq2(v1.4.5) with a Q value ≤0.05. Gene Ontology (GO) and Kyoto Encyclopedia of Genes and Genomes (KEGG) pathway enrichment analysis of the annotated DEGs were performed using Phyper, and a rigorous threshold of Q value ≤0.05 was used to correct the significant levels of terms and pathways by Bonferroni.

### Drug screening

The CCK8 assay was used to evaluate the inhibition efficiency of the marketed anti-cancer drug library against the control and PRR15-knockdown MDA-MB-231 cells. In brief, 5 different concentrations were prepared for each compound, starting at 30 µM and diluted in a 5-fold gradient, with the working final concentration of 30, 6, 1.2, 0.24 and 0.048 µM. The positive control staurosporine was used at 10 µM. Additionally, wells without cells but containing medium were used as blank controls and wells with cells and containing vehicles were used as negative controls. The percentage of inhibition was calculated as follows: inhibition % = (1-(OD_S_-OD_BLK_)/(OD_NC_-OD_BLK_))×100, where OD_S_ represents the absorbance of the samples, OD_NC_ indicates the absorbance of the negative control, and OD_BLK_ denotes the absorbance of the blank control.

### Clinical data

A total of 113 medical records from female patients diagnosed with TNBC who underwent surgery were retrospectively reviewed and collected. The sample size was determined as the appropriate number of included patients that ensured adequate statistical power when the significance level was set at α = 0.05. These patients were histologically confirmed as ER-negative, PR-negative, and HER2-negative and harbored high-risk factors for recurrence, such as age <35 years, positive axillary lymph nodes, grade III disease, or intravascular cancer embolus. The critical demographic and clinicopathological characteristics were assessed along with follow-up information. Disease-free survival (DFS) was estimated as the interval from surgery to the first local/distant recurrence or death of any cause. Overall survival (OS) was defined as the period from surgery to death due to any cause. Participants without progression, recurrence, metastasis, and death were considered as censoring events at the end of the follow-up. Informed consent and ethical approval were provided, and all data presented in the research were deprived of patient-identifying parameters.

### Bioinformatics

The transcriptomic data from The Cancer Genome Atlas (TCGA, http://cancergenome.nih.gov/), Oncomine (http://www.oncomine.org), and Genotype-Tissue Expression (GETx, https://gtexportal.org/home/) were used to analyze PRR15 expression in normal and tumor tissues of breast and other types of cancer. Gene Expression Profiling Interactive Analysis v2 (GEPIA2, http://gepia2.cancer-pku.cn) was used for pan-cancer survival analysis [16]. The information stored in Cancer Cell Line Encyclopedia (CCLE, https://portals.broadinstitute.org/ccle) was downloaded for the comparison of PRR15 expression in luminal and TNBC/basal breast cancer cells as well as for pan-cancer analysis according to previous reports [17]. Single-cell RNA sequencing (scRNA-seq) information stored in the Single Cell Portal database was used to investigate the abundance of single-cell PRR15 expression in different subtypes of breast cancer (https://singlecell.broadinstitute.org/single_cell) [18]. The number of cell types involved: Cancer Basal SC, 4312 points; Cancer Cycling, 5359 points; Cancer HER2 SC, 3708 points; Cancer LumA SC, 7742 points; Cancer LumB SC, 3368 points. The PRR15 genomic alteration in pan-cancer was detected via Gene Set Cancer Analysis (GSCA, http://bioinfo.life.hust.edu.cn/GSCA/#/). In addition to online tools, other bioinformatic analyses were performed using the R software v4.1.2.

### Statistical analysis

Statistical analysis was performed using SPSS v23.0 or GraphPad Prism v7. Cellular and molecular experiments were carried out in triplicates and at least three independent repeats were performed. Results were presented as mean ± SEM for continuous variables, and number (%) for categorical variables, unless otherwise noted. Differences between two groups were determined by unpaired *t*-test, and one-way ANOVA was used for multiple comparisons. Continuous data that did not meet the assumptions of the parametric test were evaluated using appropriate non-parametric tests. Frequency tables were examined using the χ2 test, Fisher’s exact tests, or Wilcoxon rank-sum test, as appropriate. Kaplan–Meier survival curves were evaluated with log-rank test and correlations were calculated using Spearman correlation coefficients. All statistical tests were two-tailed and a value of *P* < 0.05 was considered statistically significant.

## Results

### PRR15 expression is decreased in TNBC but increased in non-TNBC, and predicts a more favorable prognosis in the former

**Fig. 1:**
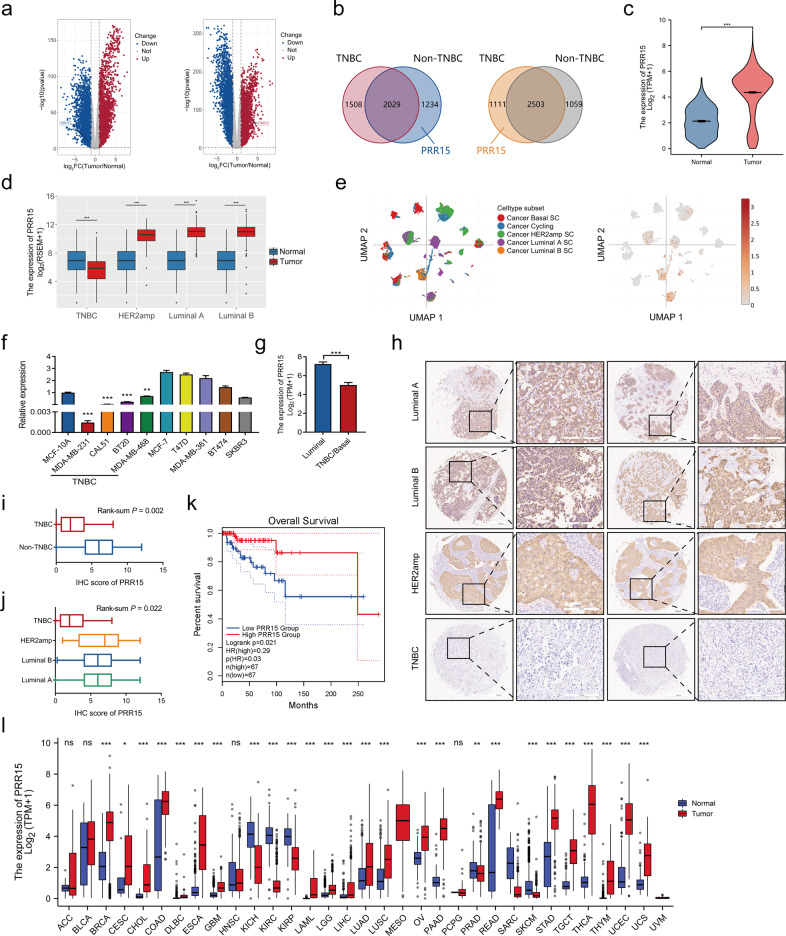
PRR15 expression is decreased in TNBC but increased in non-TNBC, and predicts a more favorable prognosis in the former. **a** DEGs in TNBC (left panel) and non-TNBC (right panel) relative to normal breast tissues. Data analyzed from TCGA and GTEx database, *P* value <0.05, |Log_2_-FC | > 2. **b** The overlap of the up-regulated genes (left panel) and down-regulated genes (right panel) of TNBC and non-TNBC obtained in Fig. 1a. **c** Transcriptomic PRR15 expression in the overall breast cancer and normal samples from TCGA database. **d** PRR15 expression in distinct molecular subtypes of breast carcinoma *versus* normal mammary epithelium. **e** PRR15 expression in different subnets of breast neoplasia identified by single-cell transcriptomics archived in the Single Cell Portal project. Left panel, quantity and distribution of subpopulations of different cell types; right panel, expression of PRR15. **f** RT-qPCR of PRR15 in a panel of cell lines. **g** PRR15 expression in luminal (*n* = 26) and TNBC/basal (*n* = 27) breast neoplasm cell lines available in CCLE. **h** PRR15 expression examined by IHC in breast cancer tissue microarrays, which included 128 cases of primary breast cancer lesions, including 24 cases of TNBC and 100 cases of non-TNBC (35 cases of luminal A, 54 cases of luminal B, 7 cases of HER2amp, and 4 cases unknown). **i**, **j** Statistical analysis of the differences in PRR15 expression between TNBC and non-TNBC (**i**) and among four molecular subsets of breast cancer (**j**) in tissue microarrays from Fig. 1h. **k** Effect of PRR15 expression on OS in TNBC as shown by Kaplan–Meier survival curve using the TCGA data. High and low PRR15 expression was discriminated using a 50% (median) cut-off value. **l** Differential PRR15 mRNA expression between multiple tumors and corresponding normal samples determined using the RNA-seq data from TCGA in combination with GTEx consortium. Data are presented as mean ± SEM (**c**, **d**, **f**, **g**) or median and interquartile range (**i**, **j**, **l**), and analyzed by unpaired *t*-test (**c**, **d**, **f**, **g**) or Wilcoxon rank-sum test (**i**, **j**, **l**). ^*^P < 0.05, ^**^P < 0.01,^***^P < 0.001. Scale bars: **h** left 500 μm, right 200 μm. *DEGs* differentially expressed genes, *TCGA* The Cancer Genome Atlas, *GTEx* Genotype-Tissue Expression, *TNBC* triple-negative breast cancer, *CCLE* Cancer Cell Line Encyclopedia, *RT-qPCR* quantitative reverse transcription polymerase chain reaction, *IHC* immunohistochemistry, *OS* overall survival.

We investigated the available data archived in TCGA and GTEx databases to gain insight into the DEGs responsible for the differences in the malignant behavior between TNBC and non-TNBC. The volcano plots in Fig. 1a show the DEGs in TNBC and non-TNBC *versus* normal breast samples, and then the intersection of the screened highly expressed genes as well as the lowly expressed genes were considered (Fig. 1a, b). Although the expression of PRR15 was significantly higher in the overall breast cancer than in the normal counterparts, the stratified analysis based on molecular typing revealed that PRR15 expression was substantially increased in luminal A, luminal B and HER2amp malignancies than in normal tissues, whereas the opposite was found in TNBC (Fig. 1c, d). Likewise, the analysis of scRNA-seq data from breast cancer supported this finding, with non-TNBC subnets, including luminal A, luminal B, and HER2amp, possessing higher PRR15 expression relative to TNBC (Fig. 1e). Subsequently, we detected PRR15 mRNA expression using RT-qPCR assay in a panel of mammary epithelial and breast cancer cells to probe the association of PRR15 with breast cancer. The non-transformed mammary epithelial cell MCF10A showed an intermediate expression of PRR15 than all the other 9 breast cancer cells (Fig. 1f). Furthermore, PRR15 expression was much lower in TNBC cells than in MCF10A and less aggressive/metastatic luminal-like, together with HER2amp breast cancer cells, which was further validated using CCLE panel (Fig. 1f, g). The analysis of the transcriptomic data from breast cancer cell lines archived in the CCLE also revealed that PRR15 was significantly less expressed in basal/TNBC cell lines than in luminal cell lines (Fig. 1g, Table S2). IHC staining of breast cancer tissue microarray suggested that the percentage and intensity of PRR15-positive cells were lower in TNBC than in non-TNBC (Fig. 1h–j). In addition, the clinical information from TCGA suggested that increased PRR15 predicted superior OS with a log-rank *P* of 0.021 and was a favorable prognostic factor for OS with the hazard ratio of 0.29 (*P* = 0.03) in TNBC patients (Fig. 1k). However, difference in OS due to PRR15 expression was not observed in the overall or other three subtypes of breast cancer (Fig. S1a–d). These findings demonstrated that PRR15 expression was low in TNBC but was high in non-TNBC, improving the outcome of TNBC.

Pan-cancer analysis was performed since the studies available on the role of PRR15 in tumors are scarce. The combination of the comparable data of TCGA and GTEx revealed that PRR15 mRNA expression was increased in 70% of tumors than in the corresponding normal adjacent tissues (Fig. 1l). However, a significant difference in its expression was found in 9 out of 23 tumors when TCGA data alone were considered (Fig. S2a). According to the Oncomine program, PRR15 expression was high in breast, esophageal, head and neck, myeloma, and pancreatic cancers (Fig. S2b). PRR15 expression also varied in a series of cell lines from CCLE dataset, with the top three being colorectal, pancreatic, and gastric cancer cell lines (Fig. S2c). Afterwards, we depicted the copy number variation (CNV) landscape of PRR15 in various neoplasms (Fig. S2d). A remarkable positive correlation was observed between PRR15 CNV and mRNA expression in 11 tumor types, such as ovarian serous cystadenocarcinoma, breast invasive carcinoma (BRCA), and low-grade glioma (LGG, Fig. S2e). Specifically, PRR15 expression in breast cancer was positively correlated with CNV of non-TNBC (*P* = 0.0013), but not with TNBC (Fig. S2f). PRR15 methylation profiles in pan-cancer were explored, revealing that PRR15 methylation degrees were markedly reduced in several tumors than in normal tissues, including BRCA, uterine corpus endometrial carcinoma, and prostate adenocarcinoma (Fig. S2g). PRR15 methylation was negatively linked to mRNA expression in 16 tumors, including BRCA, but it exhibited a positive relationship in testicular germ cell tumor (Fig. S2h). Furthermore, a substantial association between PRR15 expression and increased methylation level in TNBC was found (*P* = 0.0049), in contrast to a weak negative trend in non-TNBC but the difference was not statistically significant (Fig. S2i). Additionally, high PRR15 expression was linked to better OS in thyroid carcinoma but worse OS in LGG, lung adenocarcinoma, and pancreatic carcinoma (Fig. S3a–d).

### PRR15 silencing is required for TNBC initiation and progression in vitro and in vivo

**Fig. 2:**
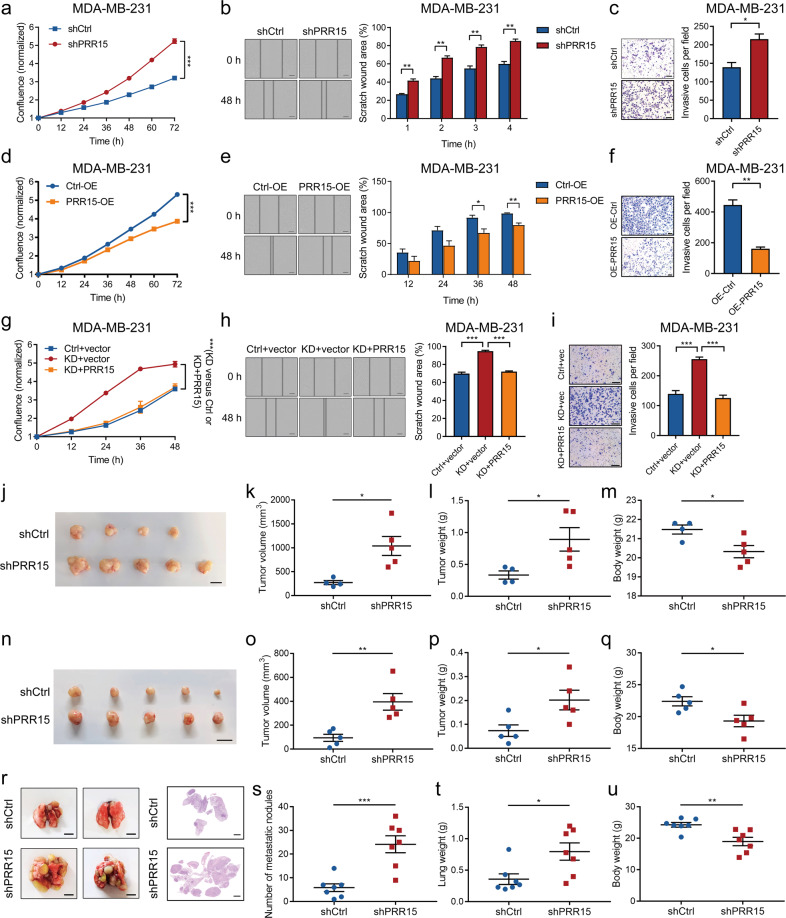
PRR15 silencing is required for TNBC initiation and progression in vitro and in vivo. **a** Proliferation of MDA-MB-231 with stable knockdown of PRR15 and scramble control monitored by the IncuCyte system. **b** Migration ability of PRR15-silenced MDA-MB-231 compared to control cells assessed by wound-healing scratch assays. **c** Effect of PRR15 knockdown on the invasive ability of MDA-MB-231 investigated using the transwell invasion assays. **d** Comparison of the proliferative ability of MDA-MB-231 stably overexpressing PRR15 with control cells by IncuCyte platform. **e** Cell motility of MDA-MB-231 with ectopic PRR15 and counterparts determined through wound-healing scratch assays. **f** Effect of PRR15 overexpression on the invasive ability of MDA-MB-231 examined using the transwell invasion assays. **g** Cell proliferation of PRR15-silenced MDA-MB-231 with or without PRR15 restoration, as well as that of their control cells assessed using Incucyte technology. **h** Comparison of the scratch healing rate in control, PRR15-knockdown, and PRR15-restored MDA-MB-231. **i** Analysis of the invasive potential in the control, PRR15-knockdown, and PRR15-restored MDA-MB-231 using the transwell invasion assay. **j–q** Representative gross images (**j**, **n**), volume (**k**, **o**), and weight (**l**, **p**) of xenograft tumors formed by PRR15-silenced MDA-MB-231 and CAL51 and their controls subcutaneously implanted into Balb/c nude mice (*n* = 4–5), along with the weight of tumor-bearing mice (**m**, **q**) described in Figs. 2j, n. **r** Representative bright-field (left panel) and H&E-stained (right panel) images of lungs from Balb/c-nude mice after the intravenous injection of PRR15-silenced MDA-MB-231 and counterparts (*n* = 7). **s–u** Number of metastatic nodules (**s**), lung weight (**t**), and body weight (**u**) of mice carrying lung metastatic lesions. Scale bars: **b** 200 μm, **c** 100 μm, **e** 200 μm, **f** 100 μm, **h** 200 μm, **i** 200 μm; **j**, **n**, 10 mm, **r** left 5 mm, right 2 mm. Data are presented as mean ± SEM, and analyzed by unpaired *t*-test. ^*^P < 0.05, ^**^P < 0.01, ^***^P < 0.001. *H&E* hematoxylin and eosin.

We determined the loss- and gain-of-function effects of PRR15 on the aggressive ability of breast cancer cells to investigate the biological role of PRR15 in tumor progression. PRR15 knockdown or overexpression was generated in breast cancer cells, including TNBC (MDA-MB-231 and CAL51) and non-TNBC (MCF7 and T47D) cells (Fig. S4a–g). The expression of the proliferation markers Ki-67 and PCNA was significantly increased in PRR15-silenced TNBC cells, and these cells displayed a remarkably increased cell growth and proliferation (Fig. S5a, b, Fig. 2a, Fig. S5c). Moreover, PRR15 downregulation resulted in an accelerated wound healing of TNBC cells, suggesting an enhanced cell mobility (Fig. 2b, Fig. S5d). More PRR15-silenced TNBC cells were found adhering to the bottom side of the membrane, indicating that PRR15 silencing exacerbated the invasive ability (Fig. 2c, Fig. S5e). In contrast, ectopic PRR15 substantially reduced Ki-67 and PCNA expression and attenuated the proliferation, motility, and invasion ability of TNBC cells (Fig. S5f–j, Fig. 2d–f, Fig. S5h-j). Next, we confirmed the observed phenotypes using a genetic rescue approach. PRR15 expression in PRR15-silenced TNBC cells was restored, resulting in the abolished potentiation of growth, migration and invasion of PRR15-silenced MDA-MB-231 and CAL51 cells, further confirming the biological functions regulated by PRR15 (Fig. 2g–i, Fig. S5k–m). Intriguingly, the alteration of PRR15 expression in non-TNBC cells did not significantly affect their oncogenic and invasive properties (Fig. S6a–g).

Thereafter, we subcutaneously injected MDA-MB-231 cells with PRR15 knockdown and scramble control into Balb/c-nude mice to assess the effect of PRR15 on the growth and metastasis of TNBC in mice. The experimental group showed a pronounced increase in the volume and weight of the xenografts and an evident decline in the body weight compared to the control group (Fig. 2j–m). Moreover, PRR15 silencing allowed the growth of transplanted tumors formed by CAL51 cells, consistent with previous findings of statistically significant differences in tumor volume and weight, as well as mouse body weight between the two groups (Fig. 2n–q). Subsequently, we injected MDA-MB-231 cells with or without PRR15 knockdown into the tail vein of Balb/c-nude mice to obtain a lung metastasis model. PRR15 knockdown induced a remarkable increase in the number and volume of visible metastatic nodules in the lungs, and H&E-stained sections revealed that the mice carrying PRR15-silenced MDA-MB-231 cells had more malignant metastatic lesions (Fig. 2r, s). In addition, the experimental mice showed an increased lung weight but a lower body weight, which might be related to cachexia caused by metastasis (Fig. 2t, u). In contrast, PRR15 knockdown did not exacerbate the initiation and progression of non-TNBC MCF7 cells in NOG mice, and no marked differences were observed between PRR15-silenced xenografts and controls in terms of tumor volume, tumor weight, and mouse body weight (Fig. S6h-k). Taken together, these data provided solid proofs that PRR15 silencing increased tumorigenesis and metastasis of TNBC rather than non-TNBC and the increase in PRR15 expression suppressed the malignant phenotypes in TNBC.

### Screening of more potent antineoplastic drugs effective on TNBC cells with decreased PRR15 expression using high-throughput drug sensitivity testing

**Fig. 3:**
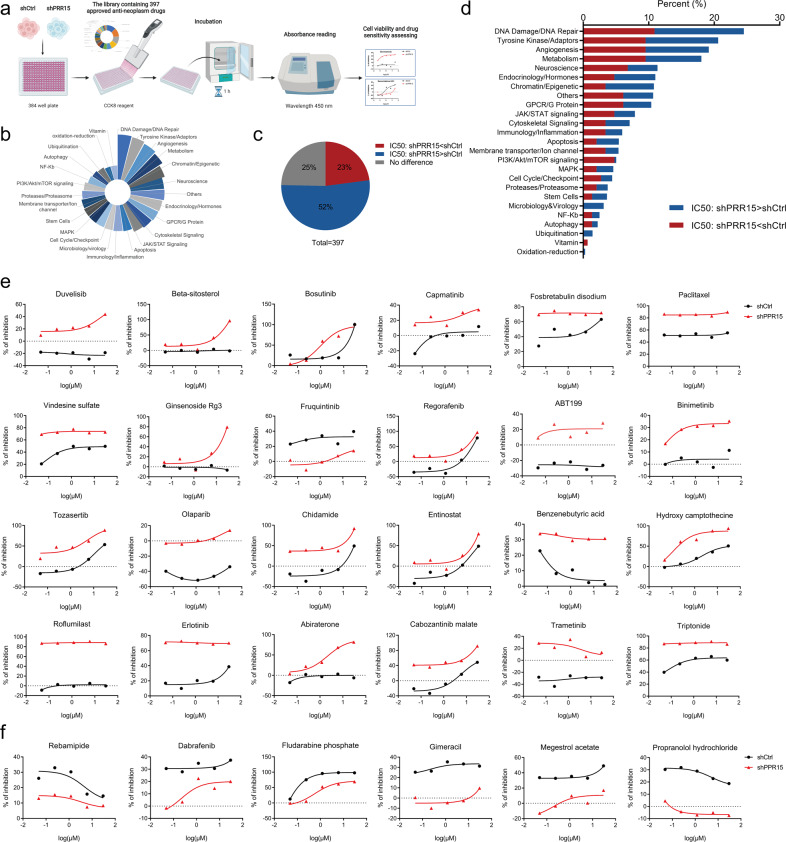
Screening of more potent antineoplastic drugs effective on TNBC cells with decreased PRR15 expression using high-throughput drug sensitivity testing. **a** Schematic diagram of a high-throughput drug screen using a library of 397 marketed anti-cancer drugs. MDA-MB-231 cells with or without PRR15 impairment treated with the indicated compounds for 72 h and cell viability measured by CCK8 assay. **b** Composition of the pathways targeted by the agents included in the library of antineoplastics. **c** Changes in IC50 values of the tested drugs on MDA-MB-231 cells after PRR15 knockdown. **d** Distribution of the molecular pathways targeted by the drugs with altered IC50 values in the Fig. 3c. **e**, **f** Representative antineoplastic agents with enhanced (i.e., reduced IC50, **e**) and attenuated (i.e., increased IC50, **f**) inhibitory effects on PRR15-silenced MDA-MB-231 cells. *IC50* half-maximal inhibitory concentration.

Since the previous results established that PRR15 silencing enhanced the formation and progression of TNBC, we addressed its effect on drug sensitivity. A library composed of 397 listed antitumor agents combined with CCK8 cytotoxicity assays was used to identify robust anticancer agents that could restrain the effect of PRR15 knockdown in TNBC (Fig. 3a). The molecular mechanism of the drug library utilized covered 25 signaling pathways, and the three pathways with the most antineoplastic targets were DNA damage/DNA repair, tyrosine kinase/ adaptors and angiogenesis pathway (Fig. 3b). The downregulation of PRR15 expression was associated with a reduced IC50 and increased the sensitivity of MDA-MB-231 cells to 23% of the drugs (out of a total of 397 drugs), increased IC50 and enhanced the resistance to 52% of the drugs, and no change in IC50 was observed for the remaining 25% of the drugs (Fig. 3c). Notably, the summary of drug pathways whose inhibitory effect was altered by PRR15 silencing demonstrated the involvement of the PI3K/Akt/mTOR signaling and consequent effectiveness of drugs affecting it (Fig. 3d). In particular, PRR15-knockdown TNBC cells were more sensitive to several drugs, including duvelisib, beta-sitosterol, bosutinib, capmatinib, fosbretabulin disodium, paclitaxel, vindesine sulfate, ginsenoside Rg3, fruquintinib, regorafenib, ABT199, binimetinib, tozasertib, Olaparib, chidamide, entinostat, and benzenebutyric acid (Fig. 3e). However, these cells were less sensitive to other drugs, including rebamipide, dabrafenib, fludarabine phosphate, gimeracil, megestrol acetate, and propranolol hydrochloride (Fig. 3f). In conclusion, the expression of PRR15 affected the inhibitory effect of anticancer drugs on TNBC cells, which might be used to optimize clinical treatment strategies.

### PI3K/Akt pathway is a pivotal mechanism by which PRR15 deficiency promotes the progression of TNBC

**Fig. 4:**
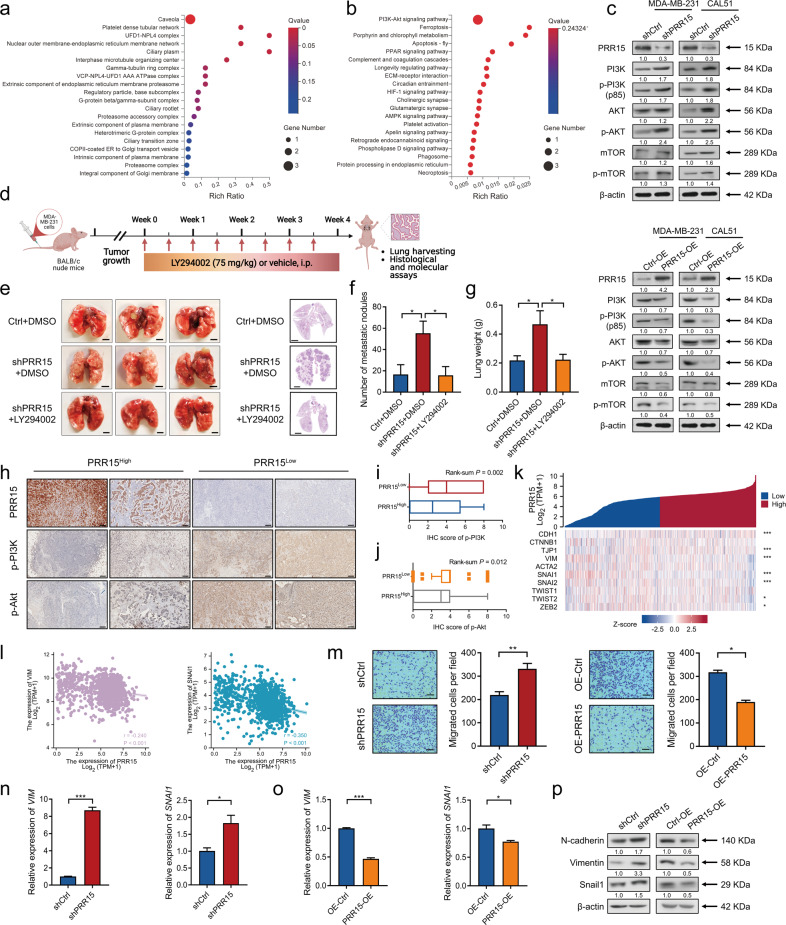
PI3K/Akt pathway is a pivotal mechanism by which PRR15 deficiency promotes the progression of TNBC. **a**, **b** Significant GO clusters for the CC categories (**a**) and KEGG pathways (**b**) enriched by the DEGs between PRR15-knockdown MDA-MB-231 cells and control cells. **c** Western blot analysis of the expression of the key factors of the PI3K/Akt signaling in MDA-MB-231 (top panel) and CAL51 (bottom panel) cells with PRR15 knockdown and overexpression. **d** Schematic representation of the study design and timeline of LY194002 treatment in mice receiving an injection of PRR15-silenced MDA-MB-231 and control cells into the tail vein. **e–g** Representative gross (**e**, left panel) and histopathological (**e**, right panel) images of the lungs of the mice mentioned in Fig. 4d, as well as the number of metastatic foci (**f**) and lung weight (**g**). **h–j** Representative images of the expression of PRR15, p-PI3K, and p-Akt in the tumor mass of TNBC patients with high and low expression of PRR15 detected by IHC (**h**), and statistics of IHC staining scores of p-PI3K (**i**) and p-Akt (**j**). **k** Co-expression heat map of PRR15 and critical molecules of EMT signaling for BRCA data analysis archived by TCGA. **l** Scatter plots of the correlation between PRR15 expression and the expression of the mesenchymal markers vimentin (*VIM*, left panel) and SNAI1 (right panel). **m** Transwell migration analysis of the indicated MDA-MB-231 cells with different expression levels of PRR15. **n**, **o** Relative expression of vimentin (*VIM*) and SNAI1 in CAL51 cells with PRR15 knockdown (**n**) and PRR15 overexpression (**o**) along with the corresponding control cells evaluated by RT-qPCR. **p** Western blot analysis of EMT markers in MDA-MB-231 cell with silencing or overexpression of PRR15 and their controls. Data are presented as mean ± SEM (**f**, **g**, **m**–**o**) or median and interquartile range (**i**, **j**), and analyzed by unpaired *t*-test (**f**, **g**, **m**–**o**), Wilcoxon rank-sum test (**i**, **j**), or Spearman correlation coefficients (**k**, **l**). ^*^P < 0.05, ^***^P < 0.001. Scale bars: **e**, left 2.5 mm, right 2 mm; **h**, 200 μm; **m**, 100 μm. *GO* Gene Ontology, *CC* Cellular Component, *KEGG* Kyoto Encyclopedia of Genes and Genomes, *DEGs* differentially expressed genes, *IHC* immunohistochemistry, *EMT* epithelial-mesenchymal transition, *BRCA* breast invasive carcinoma, *TCGA* The Cancer Genome Atlas, *RT-qPCR* quantitative reverse transcription polymerase chain reaction.

According to the above results, PI3K/Akt signaling might be responsible for the aggressive phenotype regulated by the silencing of PRR15 in TNBC. Thus, PRR15-knockdown TNBC and control cells were subjected to RNA-Seq, DEGs were obtained, and the enrichment analysis was performed to validate this result. GO enrichment analysis in the category of cellular component revealed that GO terms regarding cytoskeleton system, plasma membrane, and ciliary were significantly enriched in TNBC cells with PRR15 knockdown (Fig. 4a). Furthermore, KEGG enrichment analysis strongly suggested that the PI3K/Akt signaling might be indispensable for PRR15 to modulate the biological functions (Fig. 4b). The expression of key molecules involved in the PI3K/Akt pathway was increased in PRR15-silenced TNBC cells, implying pathway activation, which was actually the opposite in TNBC cells with ectopic PRR15 (Fig. 4c). Conversely, down-regulation of PRR15 in non-TNBC MCF7 cells decreased the expression of several molecules involved in the PI3K/Akt pathway, while the up-regulation of PRR15 increase the pathway activity (Fig. S7). The histopathologic evaluation revealed that PRR15 silencing caused the activation of PI3K/Akt signaling in xenografts and lung metastatic nodules formed by TNBC cells (Fig. S8a–c). The in vivo metastasis experiments further displayed that the treatment with the PI3K inhibitor LY294002 contributed to a dramatic reverse in the increase of TNBC lung metastasis resulting from PRR15 silencing, with a reduced histopathological malignancy, number of metastatic nodules, lung weight as well as PI3K/Akt signaling activation (Fig. 4d–g, Fig. S8d). Furthermore, the patients in our TNBC cohort were divided into PRR15^High^ and PRR15^Low^ arms based on the IHC scores. Tumor tissues with substantially decreased PRR15 expression also revealed a significantly increased p-PI3K and p-Akt expression, providing evidence on the oncogenic role of PRR15 low expression mediated by the PI3K/Akt signaling pathway (Fig. 4h–j).

The co-expression analysis of PRR15 with crucial EMT-related genes using the TCGA data revealed that several genes were markedly correlated with PRR15 (Fig. 4k). Specifically, PRR15 expression was inversely associated with the expression of the mesenchymal markers vimentin and SNAI1, and was linked to a modest increased expression of the epithelial markers E-cadherin and ZO-1, as well as with a modest reduced expression of the other mesenchymal markers SNAI2, TWIST2, and ZEB2 (Fig. 4l, Fig. S9a, b). Afterwards, we observed that PRR15 knockdown significantly enhanced the migratory performance of TNBC cells, while PRR15 overexpression reduced this ability (Fig. 4m, Fig. S10a). Moreover, we detected the critical molecules vimentin and SNAI1 in TNBC cells and we found that PRR15 silencing predominantly improved their transcript levels, while PRR15 overexpression declined their mRNA expression (Fig. 4n-o). Furthermore, western blot images and quantitative analysis showed that MD-MB-231 cells and CAL51 cells with PRR15 knockdown expression had increased levels of N-cadherin, vimentin and Snail1 proteins; the opposite was observed for PRR15-overexpressing TNBC cells (Fig. 4p, Fig. S10b), unveiling the impact of PRR15 low expression on invasion and metastasis of TNBC partly through the EMT mechanism. These results indicated that the low PRR15 expression promoted TNBC development in a PI3K/Akt pathway-dependent manner, with the involvement of EMT.

### Demographic and clinicopathological characteristics associated with PRR15 expression in TNBC patients

We retrospectively collected a total of 113 medical records along with histologically verified tumor tissue sections from TNBC patients to investigate the relationship between PRR15 expression and clinicopathological features of TNBC, and we divided the participants into two groups according to the distinct PRR15 expression as described above. The PRR15^High^ and PRR15^Low^ groups consisted of 45 and 68 patients, respectively, with an average age of 51 and 47 years. The primary factors of the enrolled patients are listed in Table 1. The comparison of the baseline characteristics of the TNBC patients in the two groups demonstrated that TNBC patients with decreased PRR15 expression were more likely to develop vascular aneurysm emboli (70.6% for yes *versus* 34.7% for no, *P* = 0.005), had a higher Ki-67 index (18.2% for Ki-67 ≤ 30 *versus* 45.1% for Ki-67 > 30, *P* = 0.021), and received radiotherapy more frequently (49.1% for yes *versus* 30.4% for no, *P* = 0.042, Table 1). Additionally, low PRR15 expression was slightly correlated with older age, although not statistically significant (28.2% for age ≤45 *versus* 45.9% for age >45, *P* = 0.067, Table 1). No other pronounced differences were observed based on pre-defined factors (Table 1). These results suggested that reduced PRR15 was associated with more aggressive clinical features in our patients with TNBC.

**Table 1:** PRR15 expression associated with clinicopathological characteristics in TNBC patients.

Characteristic	All subjects, No.	Low expression of PRR15, No. (%)	High expression of PRR15, No. (%)	*P*-value
*Age [mean (range)] (year)*	113	51 (32–63)	47 (29–64)	0.067
≤45	39	11 (28.2)	28 (71.8)	
>45	74	34 (45.9)	40 (54.1)	
*Menopause at diagnosis*				0.176
Pre-menopause	64	22 (34.4)	42 (65.6)	
Post-menopause	49	23 (46.9)	26 (53.1)	
*Comorbidity*				0.511
Yes	46	20 (43.5)	26 (56.5)	
No	67	25 (37.3)	42 (62.7)	
*Pathology*				0.193
IDC	103	39 (37.9)	64 (62.1)	
Non-IDC	10	6 (60.0)	4 (40.0)	
*Tumor size (cm)*				0.46
<2	50	18 (36.0)	32 (64.0)	
≥2	63	27 (42.9)	36 (57.1)	
*Lymph node involvement*				0.536
Yes	39	14 (35.9)	25 (64.1)	
No	74	31 (41.9)	43 (58.1)	
*Intravascular cancer embolus*				0.005
Yes	17	12 (70.6)	5 (29.4)	
No	95	33 (34.7)	62 (65.3)	
*Tumor grade*				0.592
I/II	32	14 (43.8)	18 (56.3)	
III	81	31 (38.3)	50 (61.7)	
*Ki-67 (%)*				0.021
≤30	22	4 (18.2)	18 (81.8)	
>30	91	41 (45.1)	50 (54.9)	
*TNM stage*				0.602
I	44	19 (43.2)	25 (56.8)	
II/III	68	26 (38.2)	42 (61.8)	
*Type of surgery*				0.612
MRM	85	33 (38.8)	52 (61.2)	
BCS	20	9 (45.0)	11 (55.0)	
Other	8	3	5	
*Radiotherapy*				0.042
Yes	57	28 (49.1)	29 (50.9)	
No	56	17 (30.4)	39 (69.6)	
*Chemotherapy regimen*				0.617
EC-P	56	21 (37.5)	35 (62.5)	
PC	57	24 (42.1)	33 (57.9)	

*IDC* invasive ductal carcinoma, *MRM* modified radical mastectomy, *BCS* breast conservative surgery, *EC-P* epirubicin and cyclophosphamide followed by paclitaxel, *PC* paclitaxel plus carboplatin.

### PRR15 expression acts as a robust predictor of the disease outcome in TNBC patients

**Fig. 5:**
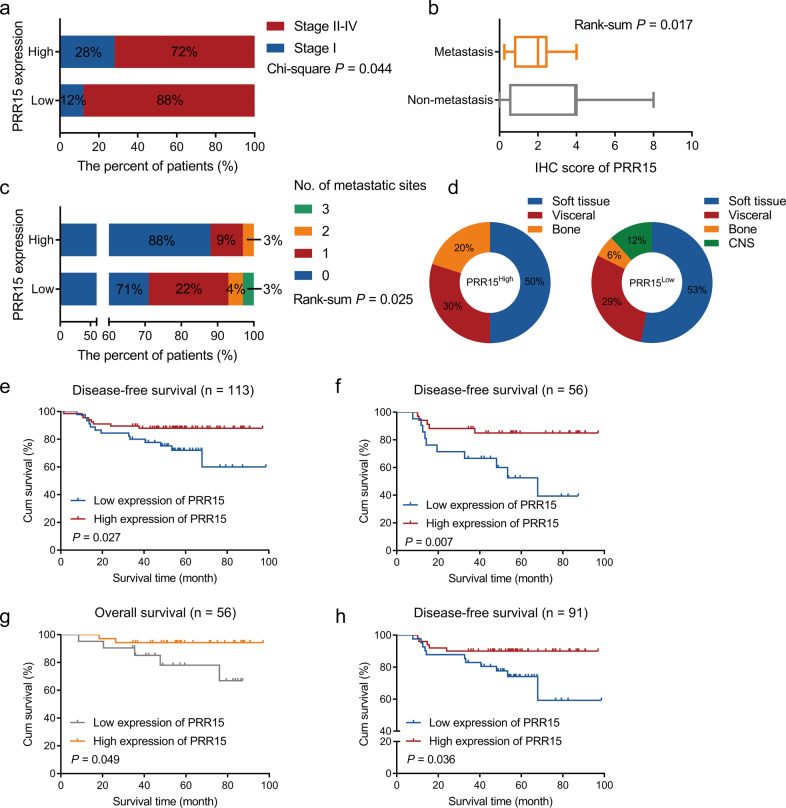
PRR15 expression acts as a robust predictor of the disease outcome in TNBC patients. **a** Comparison of clinical staging in breast cancer patients with high and low PRR15 expression using the TCGA-BRCA dataset. **b** IHC staining score of PRR15 in TNBC patients with or without metastases in the breast cancer cohort (cases corresponding to tissue microarray). **c**, **d** Statistics of the number of metastatic sites in the PRR15^High^ and PRR15^Low^ patients in our TNBC cohort (**c**) together with the profile of metastatic sites (**d**). **e–h** Kaplan–Meier survival curves for DFS in the overall TNBC cohort (**e**), for DFS (**f**) and OS (**g**) in the subgroup of TNBC patients receiving EC-P treatment, and for DFS in the subgroup of TNBC patients with high Ki-67 index (>30, **h**). Data are presented as percentage of the total (**a**, **c**, **d**) or median and interquartile range (**b**), and analyzed by χ2 test (**a**), rank-sum test (**b**, **c**), or log-rank test (**e**–**h**). *TCGA* The Cancer Genome Atlas, *BRCA* breast invasive carcinoma, *IHC* immunohistochemistry, *TNBC* triple-negative breast cancer, *DFS* disease-free survival, *OS* overall survival, *EC-P* epirubicin and cyclophosphamide followed by paclitaxel.

Our results made us wonder whether the function of PRR15 in maintaining the malignant properties of TNBC was also present in clinical patients, thus potentially affecting the therapeutic effect and prognosis. Hence, we conducted a further evaluation. The analysis of TCGA data revealed a remarkable association between PRR15 downregulation and higher clinical stages, and the corresponding clinical information of the constructed breast cancer tissue chip also displayed lower PRR15 expression in TNBC patients who experienced metastases (Fig. 5a, b). We then estimated the metastasis profile of our TNBC cohort and observed that the proportion of patients with metastasis in the PRR15^Low^ arm was higher than that in the PRR15^High^ arm (29% for PRR15^Low^ arm *versus* 12% for PRR15^High^ arm, *P* = 0.025), and 3% of patients had metastases in 3 different locations (Fig. 5c). The metastatic sites in the PRR15^High^ group included soft tissues, visceral, and bone location, but apart from these 3 metastatic foci, central nervous system (CNS) metastases occurred in the PRR15^Low^ group (Fig. 5d).

As regards the prognosis, in our overall cohort, TNBC patients with high PRR15 expression were characterized by a longer DFS but without striking differences in OS (Fig. 5e, Fig. S11a). Besides, we performed exploratory stratified analyses based on therapeutic approach and Ki-67 index. High PRR15 in the EC-P (epirubicin and cyclophosphamide followed by paclitaxel) treatment subgroup improved DFS and OS, while PRR15 expression in the PC (paclitaxel plus carboplatin) treatment subgroup did not affect clinical prognosis (Fig. 5f, g, Fig. S11b, c). Patients with increased PRR15 expression in the high Ki-67 subgroup had a better DFS, while no association between PRR15 expression and disease outcome was observed in the low Ki-67 subgroup (Fig. 5h, Fig. S11d). Altogether, low PRR15 expression was related to high invasiveness and metastasis in TNBC and might serve as a novel indicator for disease outcome.

## Discussion

TNBC is an aggressive and complex subtype characterized by an invasive behavior, tumoral heterogeneity, and lack of effective biomarkers, thereby leading to resistance to therapies, recurrence, and severe prognosis [19]. This study investigated the factors causing the distinct properties of TNBC and non-TNBC, and PRR15 was identified as a DEG between TNBC and non-TNBC with substantially decreased expression in TNBC than normal samples, while high levels were found in the overall breast cancer or non-TNBC compared with their counterparts, which was confirmed by several perspectives. The silencing of PRR15 enhanced cancer initiation and progression by regulating the PI3K/Akt signaling in vitro and in vivo. Moreover, the reduced PRR15 expression in TNBC patients was positively associated with more aggressive clinicopathological features, higher metastasis, and worse prognosis.

TNBC differs from non-TNBC in several aspects, including clinical presentation and treatment options. The striking characteristics of TNBC involved young age, unfavorable histopathology, high grade and proliferative index, absence of tubule formation, and high rate of metastasis in distant organs, including lung and brain [20]. Haffty et al. showed that the higher percentage of younger females is one of the more prominent differences between TNBC and non-TNBC populations, with 37% of the TNBC patients over 50 years of age at diagnosis as opposed to 55% of the non-TNBC group [21]. Besides, 80% of the patients with deleterious BRCA1 mutations were classified as TNBC, and the TNBC cohort includes a higher proportion of patients carrying strong family history of tumors as well as higher pathologic T stages [21]. Of note, women with TNBC have a substantially high propensity to experience intracranial disease [22]. Approximately 45% of patients diagnosed with advanced TNBC develop distant metastasis to CNS and/or visceral organs, with a median OS of 13.3 months [23]. Another study indicated that the TNBC subtype is significantly correlated with a shorter time between the diagnosis of metastasis and treatment for brain metastasis with radiotherapy, with a median time of 7.5 months [24]. Consistently, our TNBC cohort had a higher proportion of patients with metastases than the non-TNBC group, with more metastatic sites and brain metastases, demonstrating the metastatic ability of TNBC. Furthermore, a disparity exists between TNBC and non-TNBC in the treatment selection and efficacy responses. A meta-analysis of randomized trials suggested that the addition of capecitabine to standard chemotherapy results in a better DFS in TNBC than non-TNBC [25]. Meta-regression analysis demonstrated that capecitabine combined with standard chemotherapy is associated with an improved OS in studies involving higher proportions of TNBC patients than non-TNBC [25].

The relationship between PI3K/Akt signaling and TNBC progression is reported in many studies. Zhang et al. suggested that lncRNA SOX2‑OT induced by PAI‑1 promotes TNBC metastasis by activating the PI3K/Akt cascade [26]. Research concerning the treatment outcomes of metastatic TNBC showed a significant positive association between the therapy with PI3K/AKT/mTOR inhibitors in patients with cognate pathway abnormalities and improved progression-free survival [27]. Instead of a unique disease, TNBC is composed of multiple entities with striking histopathological, transcriptomic and genomic heterogeneity [28]. Seven clusters of TNBC were identified in a landmark study, named Basal-like 1 (BL1), basal-like 2 (BL2), immunomodulatory (IM), mesenchymal (M), mesenchymal stem-like (MSL), luminal androgen receptor (LAR), and unstable (UNS) [29]. Gene alterations of the PI3K/Akt signaling pathway (including PIK3CA mutations) have been reported in approximately 10% of TNBC cases [30]. For example, BL1 subtype exhibits increased copy number and amplification of PIK3CA and AKT2, whereas LAR malignancy is characterized by enrichment in mutations of PIK3CA and AKT1 [31]. Furthermore, the enriched understanding of TNBC heterogeneity effectively support the evolvement of therapeutic setting, tending to precision medicine and tailored therapy. Combination approaches with several inhibitors of the PI3K/Akt/mTOR pathway and androgen receptor, as well as cyclin-dependent kinase 4/6 are under investigation in several ongoing trails. On the other hand, the complex crosstalk between PI3K/Akt/mTOR signaling and other oncogenic pathways confers TNBC intrinsic resistance to targeted agents, such as the EGFR, MET, and MEK pathways [28, 32]. Reduced PRR15 expression might further serve as a biomarker to identify TNBC patients benefiting from PI3K/Akt/mTOR pathway blockade due to the context of TNBC heterogeneity, combined with the described TNBC clusters.

EMT is also a critical mechanism in the migration, invasion, and metastasis of tumor cells that lose their epithelial characteristics and are transformed into fibroblastic or mesenchymal features with loss of cell polarity, skeletal rearrangement, and increased motility and migration ability [33, 34]. Plenty of tumors such as breast, lung, hepatocellular carcinoma, and CRC with activated PI3K/Akt pathway show the induction of the EMT phenotype, thereby exacerbating their malignancy [35–38]. Our study found that PRR15 silencing might facilitate the progression of TNBC by regulating the PI3K/Akt signaling, and this result was confirmed at a molecular level, revealing that EMT was also involved. Strikingly, the PI3K/Akt pathway-related compounds duvelisib, beta-sitosterol and bosutinib, as well as the EMT-related agent capmatinib, showed stronger inhibitory effects on the proliferation of TNBC cells after PRR15 silencing.

Additionally, among the drugs that PRR15-silencing TNBC cells were more sensitive to, several ones act on the cytoskeleton and/or angiogenesis, such as fosbretabulin disodium, paclitaxel, vindesine sulfate, ginsenoside Rg3, fruquintinib, and regorafenib. Fosbretabulin disodium inhibits tubulin polymerization in tumor cells and also causes damage to the tumor vasculature [39]. Paclitaxel is one of the most commonly used broad-spectrum antineoplastic agents, which retards tumor growth by stabilizing microtubule aggregation [40]. Vindesine sulfate is a potent mitosis suppressor that binds to spindle tubulin, producing microtubule crystallization and mitosis blockade, which in turn causes cell death [41]. Ginsenoside Rg3, fruquintinib, and regorafenib are effective in repressing angiogenesis. Ginsenoside Rg3 hinders umbilical vein endothelial cell proliferation, and fruquintinib and regorafenib are highly selective small molecule inhibitors of the vascular endothelial growth factor receptor (VEGFR) family, while they also attenuate oncogenic multikinase activity such as KIT and RET [42–44]. Furthermore, TNBC patients show an increased microvascular density and intratumoral VEGF, leading to a potential beneficial effect of the therapy with bevacizumab [45–48]. Our findings, combined with the published reports mentioned above, suggested that silencing PRR15 was closely associated with increased tumor cell plasticity and vascularization activity, thereby increasing the metastatic potential of TNBC, providing a basis for the development of novel strategies for effective therapeutic interventions.

Embryogenesis development and tumor progression share the same signaling pathways and mechanisms of action, since invasive trophoblast cells have the same biological behavior as cancer cells, and both are in a hypoxic environment [49]. Interestingly, PRR15 is expressed in trophoblast cells during early gestation, and knocking down PRR15 expression in sheep embryos by shRNA leads to embryonic death or abnormal development [12]. Another study demonstrated that cyclosporine A mediates the expression of several molecules including PRR15 to promote proliferation, migration and invasion of trophoblast cells and facilitate embryo implantation [14]. Therefore, according to these discrepant results, our hypothesis was that PRR15 might exert a context-dependent effect in embryonic trophoblast cells and TNBC cells of adult individuals, which of course needs further clarification.

Several limitations are present in this study, such as the lack of detailed mechanisms used by PRR15 regulates the PI3K/Akt pathway or EMT effects. However, further research on the in-depth and direct signaling mechanisms involved will be performed in our next study. Collectively, this work was the first exploring the effect of PRR15 on TNBC using basic experiments. Our findings revealed an unexpected function of PRR15 in TNBC compared to non-TNBC by comprehensive knockdown, overexpression, and restoration (rescue) approaches using multiple in vitro and in vivo experiments. PRR15 silencing promoted the malignancy of TNBC by orchestrating PI3K/Akt signaling and altering the sensitivity of TNBC to anticancer drugs. PRR15 expression was correlated with aggressive clinicopathological features and acted as a novel prognostic biomarker of the clinical outcome in TNBC.

## Supplementary information

Supplemental Material-Supplementary Figure Legends and Tables

Figure S1

Figure S2

Figure S3

Figure S4

Figure S5

Figure S6

Figure S7

Figure S8

Figure S9

Figure S10

Figure S11

Supplemental Material-WB (revised)

Reproducibility checklist

## Data Availability

All datasets generated and analyzed during the current study are included in this submitted article (and its supplementary materials). The raw RNA-seq reads have been deposited in the Sequence Read Archive (accession number: PRJNA934262). Further inquiries can be directed to the corresponding author.

## References

[1] Wang W, Han D, Cai Q, Shen T, Dong B, Lewis MT (2022). MAPK4 promotes triple negative breast cancer growth and reduces tumor sensitivity to PI3K blockade. Nat Commun.

[2] Davies C, Pan H, Godwin J, Gray R, Arriagada R, Raina V (2013). Long-term effects of continuing adjuvant tamoxifen to 10 years versus stopping at 5 years after diagnosis of oestrogen receptor-positive breast cancer: ATLAS, a randomised trial. Lancet..

[3] Wang X, Chen T, Li C, Li W, Zhou X, Li Y (2022). CircRNA-CREIT inhibits stress granule assembly and overcomes doxorubicin resistance in TNBC by destabilizing PKR. J Hematol Oncol.

[4] Cserni G, Quinn CM, Foschini MP, Bianchi S, Callagy G, Chmielik E (2021). Triple-Negative Breast Cancer Histological Subtypes with a Favourable Prognosis. Cancers.

[5] Ribatti D, Nico B, Ruggieri S, Tamma R, Simone G, Mangia A (2016). Angiogenesis and Antiangiogenesis in Triple-Negative Breast cancer. Transl Oncol.

[6] Krause R, Hemberger M, Himmelbauer H, Kalscheuer V, Fundele RH (1999). Identification and characterization of G90, a novel mouse RNA that lacks an extensive open reading frame. Gene..

[7] Glover MD, Seidel GE (2003). Increased messenger RNA for allograft inflammatory factor-1, LERK-5, and a novel gene in 17.5-day relative to 15.5-day bovine embryos. Biol Reprod.

[8] Meunier D, Patra K, Smits R, Hägebarth A, Lüttges A, Jaussi R (2011). Expression analysis of proline rich 15 (Prr15) in mouse and human gastrointestinal tumors. Mol Carcinog.

[9] Xing S, Wang Y, Hu K, Wang F, Sun T, Li Q (2020). WGCNA reveals key gene modules regulated by the combined treatment of colon cancer with PHY906 and CPT11. Biosci Rep.

[10] Yin X, Wang P, Yang T, Li G, Teng X, Huang W (2020). Identification of key modules and genes associated with breast cancer prognosis using WGCNA and ceRNA network analysis. Aging..

[11] Um SW, Kim Y, Lee BB, Kim D, Lee KJ, Kim HK (2018). Genome-wide analysis of DNA methylation in bronchial washings. Clin Epigenetics.

[12] Purcell SH, Cantlon JD, Wright CD, Henkes LE, Seidel GE, Anthony RV (2009). The involvement of proline-rich 15 in early conceptus development in sheep. Biol Reprod.

[13] Kiba T, Kintaka Y, Suzuki Y, Ishizuka N, Ishigaki Y, Inoue S (2010). Gene expression profiling in rat pancreas after ventromedial hypothalamic lesioning. Pancreas..

[14] Huang W, Lu W, Li Q, Zhang Y, Xie B, Luo S (2020). Effects of cyclosporine A on proliferation, invasion and migration of HTR-8/SVneo human extravillous trophoblasts. Biochem Biophys Res Commun.

[15] Gates KC, Goetzmann LN, Cantlon JD, Jeckel KM, Anthony RV (2017). Effect of proline rich 15-deficiency on trophoblast viability and survival. PLoS ONE.

[16] Tang Z, Kang B, Li C, Chen T, Zhang Z (2019). GEPIA2: an enhanced web server for large-scale expression profiling and interactive analysis. Nucl Acids Res.

[17] Dai X, Cheng H, Bai Z, Li J (2017). Breast Cancer Cell Line Classification and Its Relevance with Breast Tumor Subtyping. J Cancer.

[18] Wu SZ, Al-Eryani G, Roden DL, Junankar S, Harvey K, Andersson A (2021). A single-cell and spatially resolved atlas of human breast cancers. Nat Genet.

[19] So JY, Ohm J, Lipkowitz S, Yang L (2022). Triple negative breast cancer (TNBC): Non-genetic tumor heterogeneity and immune microenvironment: Emerging treatment options. Pharmacol Ther.

[20] Irvin WJ, Carey LA (2008). What is triple-negative breast cancer?. Eur J Cancer.

[21] Haffty BG, Yang Q, Reiss M, Kearney T, Higgins SA, Weidhaas J (2006). Locoregional relapse and distant metastasis in conservatively managed triple negative early-stage breast cancer. J Clin Oncol.

[22] Kuksis M, Gao Y, Tran W, Hoey C, Kiss A, Komorowski AS (2021). The incidence of brain metastases among patients with metastatic breast cancer: a systematic review and meta-analysis. Neuro Oncol.

[23] Lin NU, Claus E, Sohl J, Razzak AR, Arnaout A, Winer EP (2008). Sites of distant recurrence and clinical outcomes in patients with metastatic triple-negative breast cancer: high incidence of central nervous system metastases. Cancer..

[24] Wang XY, Rosen MN, Chehade R, Sahgal A, Das S, Warner E (2022). Analysis of Rates of Brain Metastases and Association With Breast Cancer Subtypes in Ontario, Canada. JAMA Netw Open.

[25] Natori A, Ethier JL, Amir E, Cescon DW (2017). Capecitabine in early breast cancer: A meta-analysis of randomised controlled trials. Eur J Cancer.

[26] Zhang W, Yang S, Chen D, Yuwen D, Zhang J, Wei X (2022). SOX2-OT induced by PAI-1 promotes triple-negative breast cancer cells metastasis by sponging miR-942-5p and activating PI3K/Akt signaling. Cell Mol Life Sci.

[27] Ganesan P, Moulder S, Lee JJ, Janku F, Valero V, Zinner RG (2014). Triple-negative breast cancer patients treated at MD Anderson Cancer Center in phase I trials: improved outcomes with combination chemotherapy and targeted agents. Mol Cancer Ther.

[28] Marra A, Trapani D, Viale G, Criscitiello C, Curigliano G (2020). Practical classification of triple-negative breast cancer: intratumoral heterogeneity, mechanisms of drug resistance, and novel therapies. NPJ Breast Cancer.

[29] Lehmann BD, Bauer JA, Chen X, Sanders ME, Chakravarthy AB, Shyr Y (2011). Identification of human triple-negative breast cancer subtypes and preclinical models for selection of targeted therapies. J Clin Invest.

[30] Perou CM, Sorlie T, Eisen MB, van de Rijn M, Jeffrey SS, Rees CA (2012). Comprehensive molecular portraits of human breast tumours. Nature.

[31] Bareche Y, Venet D, Ignatiadis M, Aftimos P, Piccart M, Rothe F (2018). Unravelling triple-negative breast cancer molecular heterogeneity using an integrative multiomic analysis. Ann Oncol.

[32] Vasudevan S, Adejumobi IA, Alkhatib H, Roy Chowdhury S, Stefansky S, Rubinstein AM (2021). Drug-Induced Resistance and Phenotypic Switch in Triple-Negative Breast Cancer Can Be Controlled via Resolution and Targeting of Individualized Signaling Signatures. Cancers. Cancers (Basel).

[33] Mittal V (2018). Epithelial Mesenchymal Transition in Tumor Metastasis. Annu Rev Pathol.

[34] Aouad P, Zhang Y, De Martino F, Stibolt C, Ali S, Ambrosini G (2022). Epithelial-mesenchymal plasticity determines estrogen receptor positive breast cancer dormancy and epithelial reconversion drives recurrence. Nat Commun.

[35] Alsuliman A, Colak D, Al-Harazi O, Fitwi H, Tulbah A, Al-Tweigeri T (2015). Bidirectional crosstalk between PD-L1 expression and epithelial to mesenchymal transition: significance in claudin-low breast cancer cells. Mol Cancer.

[36] Jeon YK, Kim CK, Hwang KR, Park HY, Koh J, Chung DH (2017). Pellino-1 promotes lung carcinogenesis via the stabilization of Slug and Snail through K63-mediated polyubiquitination. Cell Death Differ.

[37] Ma XL, Shen MN, Hu B, Wang BL, Yang WJ, Lv LH (2019). CD73 promotes hepatocellular carcinoma progression and metastasis via activating PI3K/AKT signaling by inducing Rap1-mediated membrane localization of P110β and predicts poor prognosis. J Hematol Oncol.

[38] Chen X, Xu M, Xu X, Zeng K, Liu X, Pan B (2020). METTL14-mediated N6-methyladenosine modification of SOX4 mRNA inhibits tumor metastasis in colorectal cancer. Mol Cancer.

[39] Shen CH, Shee JJ, Wu JY, Lin YW, Wu JD, Liu YW (2010). Combretastatin A-4 inhibits cell growth and metastasis in bladder cancer cells and retards tumour growth in a murine orthotopic bladder tumour model. Br J Pharmacol.

[40] Xi J, Zhang Z, Wang Z, Wu Q, He Y, Xu Y (2022). Hinokitiol functions as a ferroptosis inhibitor to confer neuroprotection. Free Radic Biol Med.

[41] Bánóczi Z, Gorka-Kereskényi Á, Reményi J, Orbán E, Hazai L, Tökési N (2010). Synthesis and in vitro antitumor effect of vinblastine derivative-oligoarginine conjugates. Bioconjug Chem.

[42] Xu RH, Li J, Bai Y, Xu J, Liu T, Shen L (2017). Safety and efficacy of fruquintinib in patients with previously treated metastatic colorectal cancer: a phase Ib study and a randomized double-blind phase II study. J Hematol Oncol.

[43] Chen B, Gao A, Tu B, Wang Y, Yu X, Wang Y (2020). Metabolic modulation via mTOR pathway and anti-angiogenesis remodels tumor microenvironment using PD-L1-targeting codelivery. Biomaterials..

[44] Nakhjavani M, Smith E, Townsend AR, Price TJ, Hardingham JE (2020). Anti-Angiogenic Properties of Ginsenoside Rg3. Molecules. Molecules.

[45] Mohammed RA, Ellis IO, Mahmmod AM, Hawkes EC, Green AR, Rakha EA (2011). Lymphatic and blood vessels in basal and triple-negative breast cancers: characteristics and prognostic significance. Mod Pathol.

[46] Linderholm BK, Hellborg H, Johansson U, Elmberger G, Skoog L, Lehtiö J (2009). Significantly higher levels of vascular endothelial growth factor (VEGF) and shorter survival times for patients with primary operable triple-negative breast cancer. Ann Oncol.

[47] Burstein HJ, Elias AD, Rugo HS, Cobleigh MA, Wolff AC, Eisenberg PD (2008). Phase II study of sunitinib malate, an oral multitargeted tyrosine kinase inhibitor, in patients with metastatic breast cancer previously treated with an anthracycline and a taxane. J Clin Oncol.

[48] von Minckwitz G, Eidtmann H, Rezai M, Fasching PA, Tesch H, Eggemann H (2012). Neoadjuvant chemotherapy and bevacizumab for HER2-negative breast cancer. N Engl J Med.

[49] Murray MJ, Lessey BA (1999). Embryo implantation and tumor metastasis: common pathways of invasion and angiogenesis. Semin Reprod Endocrinol.

