# Supplementary material for: PRR15 deficiency facilitates malignant progression by mediating PI3K/Akt signaling and predicts clinical prognosis in triple-negative rather than non-triple-negative breast cancer
Source: Cell Death Dis. 2023 Apr 18;14(4):272. doi: 10.1038/s41419-023-05746-8 (PMC10113191; doi:10.1038/s41419-023-05746-8)
Supplement: Supplementary file 1 — Supplemental Material-Supplementary Figure Legends and Tables [file 41419_2023_5746_MOESM1_ESM.docx]

Supplementary information for

**PRR15 deficiency facilitates malignant progression by mediating PI3K/Akt signaling and predicts clinical prognosis in triple-negative rather than non-triple-negative breast cancer**

Fengzhu Guo^*^, Jialu Ma^*^, Cong Li^*^, Shuning Liu, Weizheng Wu, Chunxiao Li, Jiani Wang, Jinsong Wang, Zhijun Li, Jingtong Zhai, Fangzhou Sun, Yantong Zhou, Changyuan Guo✉, Haili Qian✉, Binghe Xu✉

^*^Fengzhu Guo, Jialu Ma, and Cong Li contributed equally to this work.

✉Correspondence: Binghe Xu, xubingheBM@163.com; Haili Qian, qianhaili001@163.com; Changyuan Guo, audreygcy@163.com.

**Supplementary Figure Legends**

**Fig. S1 a-d** OS of high and low PRR15 expression in patients in the overall breast cancer (**a**), luminal A breast cancer (**b**), luminal B breast cancer (**c**) and HER2amp breast cancer (**d**) as shown by Kaplan-Meier survival curve and analyzed by log-rank test using the TCGA data. High and low PRR15 expression was discriminated using a 50% (median) cut-off value.

**Abbreviations:** OS, overall survival; TCGA, The Cancer Genome Atlas.

**Fig. S2 a** Human PRR15 mRNA expression in different tumors and healthy counterparts from the TCGA dataset as assessed by TIMER. **b** Increased or decreased PRR15 expression in the data sets of different malignancies as opposed to normal tissues in the Oncomine database. **c** PRR15 transcriptome expression in various tumor cell lines archived in the CCLE database. **d-e** CNV landscape of PRR15 in various neoplasms (**d**) and the correlation between CNV and mRNA expression (**e**). **f** Correlation between PRR15 expression and CNV in TNBC and non-TNBC analyzed with TCGA data. **g-h** PRR15 methylation differences between malignant tissues and normal samples in pan-cancer (**g**) and correlation between methylation and mRNA expression (**h**). **i** Association of PRR15 expression with methylation in TNBC and non-TNBC analyzed using TCGA data. Data are presented as median and interquartile range (**a**) or mean ± SEM (**c**), and analyzed by Wilcoxon test (**a**, **g**), Kruskal-Wallis test (**c**), or Spearman correlation coefficients (**e**, **f**, **h**, **i**). **P* < 0.05, ****P* < 0.001.

**Abbreviations:** TCGA, The Cancer Genome Atlas; CCLE, Cancer Cell Line Encyclopedia; CNV, copy number variation; TNBC, triple-negative breast cancer.

**Fig. S3 a-d** OS of patients with different PRR15 expression in thyroid carcinoma (**a**), lower-grade glioma (**b**), lung adenocarcinoma (**c**), and pancreatic adenocarcinoma (**d**) obtained by the Kaplan-Meier method.

**Abbreviation:** OS, overall survival.

**Fig. S4 a-g** RT-qPCR analysis of PRR15 knockdown in the TNBC cell lines MDA-MB-231 (**a**) and CAL51 (**b**) and non-TNBC cell lines MCF-7 (**c**) and T47D (**d**) together with PRR15 overexpression in MDA-MB-231 (**e**), CAL51 (**f**), and MCF7 (**g**) cells involved in this study. Data are presented as mean ± SEM, and analyzed by unpaired *t*-test. ***P* < 0.01, ****P* < 0.001.

**Abbreviations:** RT-qPCR, quantitative reverse transcription polymerase chain reaction; TNBC, triple-negative breast cancer.

**Fig. S5 a-b** Relative expression of Ki-67 (*MKI67*, **a**) as well as PCNA (**b**) in PRR15-silenced CAL51 cells and controls determined by RT-qPCR. **c** Proliferation of CAL51 with stable knockdown of PRR15 and scramble control monitored by the IncuCyte system. **d** Migratory capacity of PRR15-silenced CAL51 compared to control cells assessed using the wound-healing assay. **e** Effect of PRR15 knockdown on the invasive ability of CAL51 investigated using the transwell invasion assay. **f-g** RT-qPCR of Ki-67 (*MKI67*, **f**) and PCNA (**g**) in ectopic PRR15-expressing CAL51 and control cells. **h** Proliferative ability of CAL51 stably overexpressing PRR15 with control cells by IncuCyte platform. **i** Cell motility of CAL51 with ectopic PRR15 and counterparts determined by the wound-healing assay. **j** Effect of PRR15 overexpression on the invasive ability of CAL51 examined using the transwell invasion assay. **k** Cell proliferation of PRR15-silenced CAL51 with or without PRR15 restoration as well as their control cells assessed using Incucyte technology. **l** Wound healing rate in the control, PRR15-knockdown, and PRR15-restored CAL51. **m** Analysis of the invasive potential in the control, PRR15-knockdown, and PRR15-restored CAL51 using transwell invasion assay. Data are presented as mean ± SEM, and analyzed by unpaired *t*-test. **P* < 0.05, ***P* < 0.01, ****P* < 0.001. Scale bars: d, 200 μm; e, 100 μm; i, 200 μm; j, 100 μm; l, 200 μm; m, 200 μm.

**Abbreviations:** PCNA, proliferating cell nuclear antigen; RT-qPCR, quantitative reverse transcription polymerase chain reaction.

**Fig. S6 a-b** Proliferation of MCF7 (**a**) and T47D (**b**) with PRR15 knockdown or scramble control examined by IncuCyte platform. **c-d** Effect of PRR15 silencing on the migratory ability of MCF7 (**c**) and T47D (**d**) cells in the wound healing assay. **e** Assessment of the invasive ability of PRR15-silenced and control MCF7 cells by the transwell invasion assay. **f-g** Changes in the migratory (**f**) and invasive (**g**) ability of MCF7 cells due to PRR15 overexpression. **h-k** Representative gross images (**h**), volume (**i**), and weight (**j**) of xenograft tumors formed by PRR15-silenced MCF7 cells and controls subcutaneously implanted into NOG mice (n = 6), along with the weight of tumor-bearing mice (**k**) described in Fig. S6h. Data are presented as mean ± SEM, and analyzed by unpaired *t*-test. ns: not significant. Scale bars: c, 200 μm; d, 200 μm; e, 100 μm; f, 200 μm; g, 100 μm; h, 1 cm.

**Fig. S7** Western blot analysis of the expression of the key factors of the PI3K/Akt signaling in MCF7 cells with PRR15 knockdown and overexpression.

**Fig. S8** **a-d** Representative images of IHC staining of p-PI3K and p-Akt in xenografts obtained by the inoculation of MDA-MB-231 (**a**) and CAL51 (**b**) cells with PRR15 knockdown, in lung metastatic foci formed by MDA-MB-231 with PRR15 knockdown (**c**), and in metastatic lung nodules formed by PRR15-silenced MDA-MB-231 treated with the PI3K inhibitor LY294002 (**d**), as well as their control cells. Scale bar, 100 μm.

**Fig. S9** **a-b** Scatter plots of the correlation between PRR15 expression and the expression of the epithelial markers (**a**) E-cadherin (*CDH1*) and ZO-1 (*TJP1*) and mesenchymal markers (**b**) SNAI2, TWIST2, and ZEB2. Data are analyzed by Spearman correlation coefficients.

**Fig. S10** **a** Transwell migration analysis of the indicated CAL51 cells with different expression levels of PRR15. **b** Western blot analysis of EMT markers in CAL51 cell with silencing or overexpression of PRR15 and their controls. Data are presented as mean ± SEM (**a**), and analyzed by unpaired *t*-test (**a**). **P* < 0.05, ****P* < 0.001. Scale bars: a, 100 μm.

**Abbreviation:** EMT, epithelial–mesenchymal transition.

**Fig. S11 a** OS of PRR15^High^ and PRR15^Low^ groups in our TNBC patients as shown by the Kaplan-Meier survival curve. **b-c** DFS (**b**) and OS (**c**) according to PRR15 expression in a subgroup of TNBC patients receiving PC regimen, as shown by the Kaplan-Meier survival curve. **d** DFS analysis based on PRR15 expression in a subgroup of TNBC patients with Ki-67 index ≤30 as shown by the Kaplan-Meier survival curve. Data are analyzed by log-rank test.

**Abbreviations:** OS, overall survival; TNBC, triple-negative breast cancer; DFS, disease-free survival; PC, paclitaxel plus carboplatin.

**Table S1** Sequences of shRNAs and primers involved in this study.

| **Category** | **Name** | **Sequence** |
| --- | --- | --- |
| shRNA for PRR15 knockdown | shPRR15-F | ccgggtTGTCTCATTCCACCAAATTCTCGAGAATTTGGTGGAATGAGACAACTTTTTg |
|  | shPRR15-R | aattcaaaaagtTGTCTCATTCCACCAAATTCTCGAGAATTTGGTGGAATGAGACAAC |
|  | Scramble | TTCTCCGAACGTGTCACGT |
| Sequence for PRR15 overexpression | DNA fragment encoding PRR15 | ATGGCCGACAGCGGCGATGCTGGCAGCTCCGGCCCCTGGTGGAAATCGCTCACCAACAGCAGAAAGAAAAGCAAGGAAGCCGCAGTGGGGGTGCCGCCTCCCGCCCAGCCCGCTCCCGGGGAGCCCACGCCACCTGCGCCGCCCAGCCCGGACTGGACCAGCAGCTCCCGGGAGAACCAGCACCCCAATCTCCTCGGGGGCGCCGGCGAGCCCCCCAAACCAGACAAGTTATACGGGGACAAATCCGGCAGCAGCCGCCGCAATTTGAAGATCTCGCGCTCCGGCCGCTTTAAGGAGAAGAGGAAAGTGCGCGCCACGCTGCTCCCGGAGGCGGGCAGGTCCCCGGAGGAGGCAGGCTTTCCTGGTGACCCCCACGAGGACAAGCAGTAG |
| Primers | PRR15-F | CCGCCGCAATTTGAAGAT |
|  | PRR15-R | GGGGTCACCAGGAAAGCC |
|  | MKI67-F | CTACTAAAATGCCCTGCGAATC |
|  | MKI67-R | TCCTCACCTCCTGGTACTTTATCT |
|  | PCNA-F | GGCACTCAAGGACCTCATCA |
|  | PCNA-R | GCAGCGGTAGGTGTCGAA |
|  | SNAI1-F | TTTACCTTCCAGCAGCCCTAC |
|  | SNAI1-R | GCCTTTCCCACTGTCCTCAT |
|  | VIM-F | AAATGGCTCGTCACCTTCG |
|  | VIM-R | TGGGTATCAACCAGAGGGAGT |
|  | GAPDH-F | TGGGTGTGAACCATGAGAAGT |
|  | GAPDH-R | TGAGTCCTTCCACGATACCAA |
|  | ACTIN-F | AAGGTTTCGGCGGCAAATAC |
|  | ACTIN-R | TTCTGGGACTCGTGCTTCTC |

**Table S2** Luminal and basal/TNBC cell lines and their PRR15 expression levels from the CCLE database.

| **Cell line (Luminal)** | **PRR15 expression**  **Log_2_ (TPM+1)** | **Cell line (Basal/TNBC)** | **PRR15 expression**  **Log_2_ (TPM+1)** |
| --- | --- | --- | --- |
| UACC812 | 6.608323 | BT549 | 3.714663 |
| UACC893 | 7.954302 | HMEL | 3.72613 |
| YMB1 | 8.096215 | HS739T | 3.789434 |
| MDAMB361 | 7.91203 | HS578T | 3.886953 |
| EFM192A | 7.265339 | CAL120 | 3.902485 |
| EVSAT | 5.863338 | HCC1569 | 3.930086 |
| BT474 | 8.167674 | HCC1395 | 3.945086 |
| HCC2218 | 7.761924 | MDAMB157 | 3.987408 |
| BT483 | 7.096833 | DU4475 | 4.000492 |
| ZR7530 | 7.84517 | CAL148 | 4.068286 |
| JIMT1 | 6.419007 | HCC1599 | 4.09529 |
| MDAMB175VII | 8.702648 | CAL51 | 4.194328 |
| HMC18 | 4.27721 | HCC2157 | 4.233141 |
| T47D | 5.144546 | MDAMB231 | 4.25428 |
| ZR751 | 7.414404 | HCC38 | 4.343022 |
| HCC1419 | 8.846518 | MDAMB436 | 4.851891 |
| MDAMB453 | 7.557776 | HCC1806 | 5.289879 |
| MCF7 | 6.361892 | HCC1187 | 5.391392 |
| MDAMB415 | 7.326663 | HCC1143 | 5.414796 |
| EFM19 | 4.879411 | MDAMB468 | 6.002325 |
| AU565 | 7.764315 | HCC70 | 6.057646 |
| MDAMB134VI | 8.362142 | HCC1954 | 6.377306 |
| HCC202 | 7.836645 | CAL851 | 6.79452 |
| SKBR3 | 8.976743 | HCC1937 | 6.830598 |
| CAMA1 | 7.004629 | BT20 | 7.188722 |
| HCC1428 | 6.538963 | KPL1 | 7.439611 |
|  |  | HCC1500 | 7.66851 |

**Abbreviation:** CCLE, Cancer Cell Line Encyclopedia.
