# Supplementary figures and images for: PRR15 deficiency facilitates malignant progression by mediating PI3K/Akt signaling and predicts clinical prognosis in triple-negative rather than non-triple-negative breast cancer
Source: Cell Death Dis. 2023 Apr 18;14(4):272. doi: 10.1038/s41419-023-05746-8 (PMC10113191; doi:10.1038/s41419-023-05746-8)

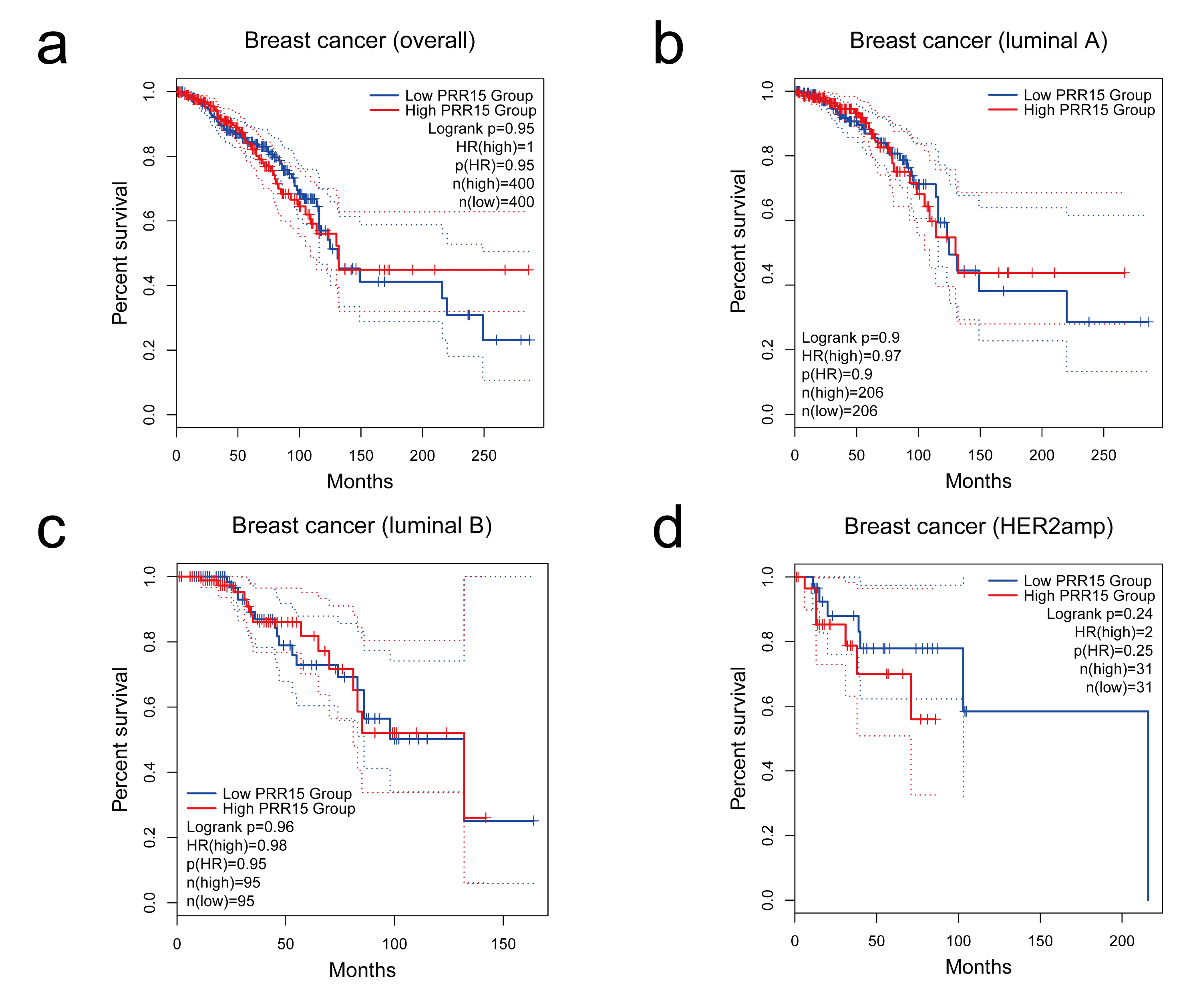

Supplement: Supplementary file 2 — Figure S1 [file 41419_2023_5746_MOESM2_ESM.tif]

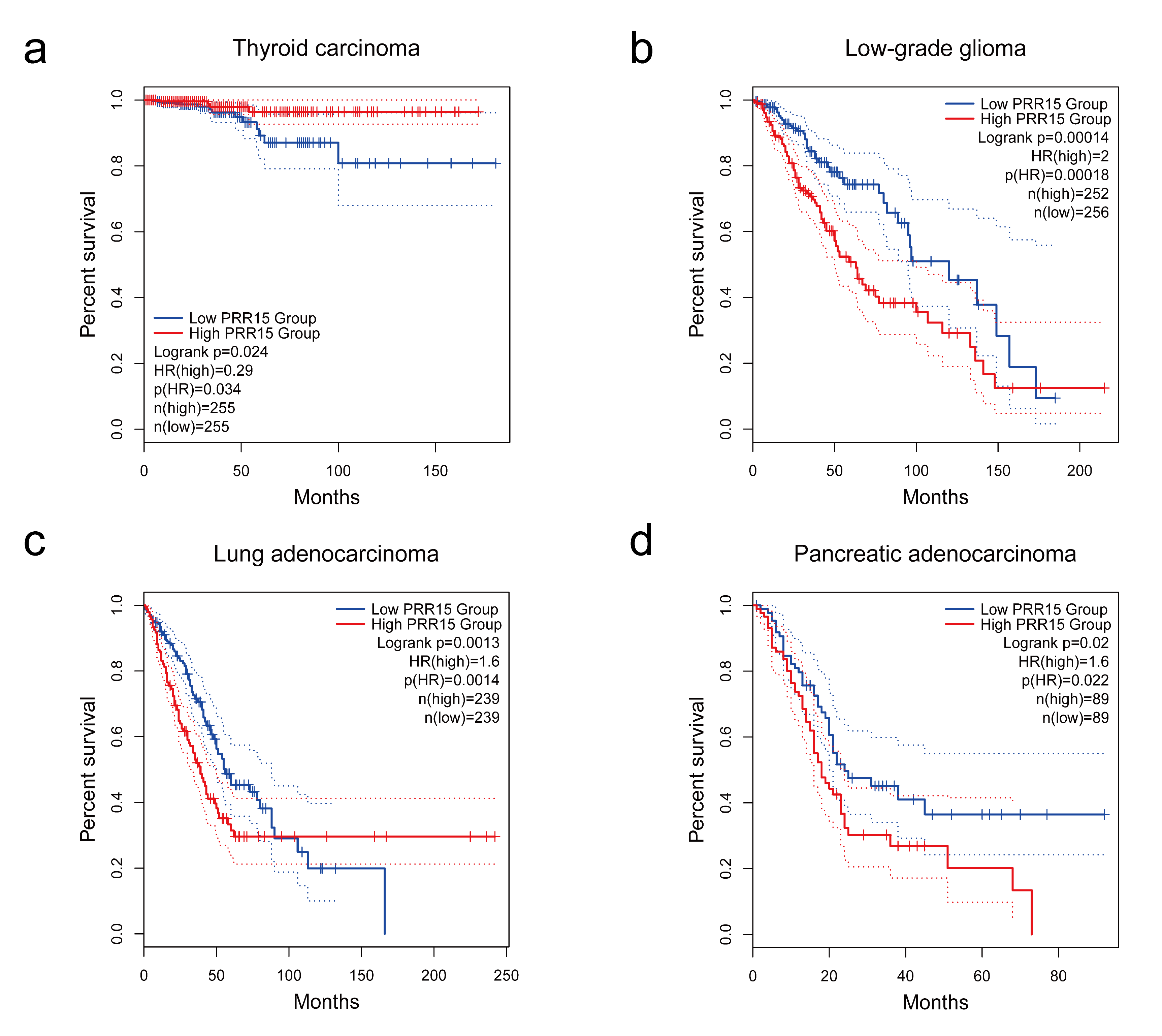

Supplement: Supplementary file 4 — Figure S3 [file 41419_2023_5746_MOESM4_ESM.tif]

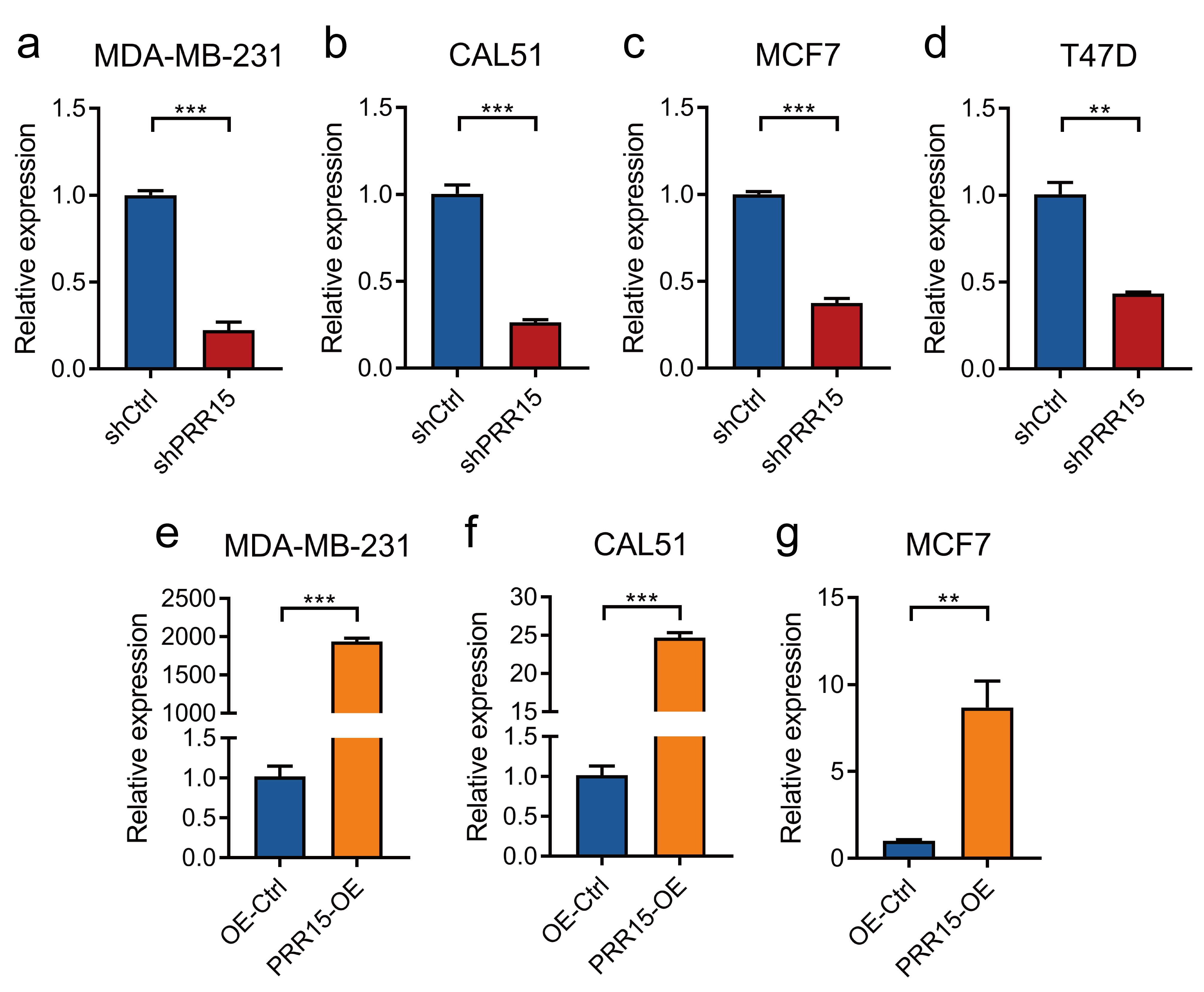

Supplement: Supplementary file 5 — Figure S4 [file 41419_2023_5746_MOESM5_ESM.tif]

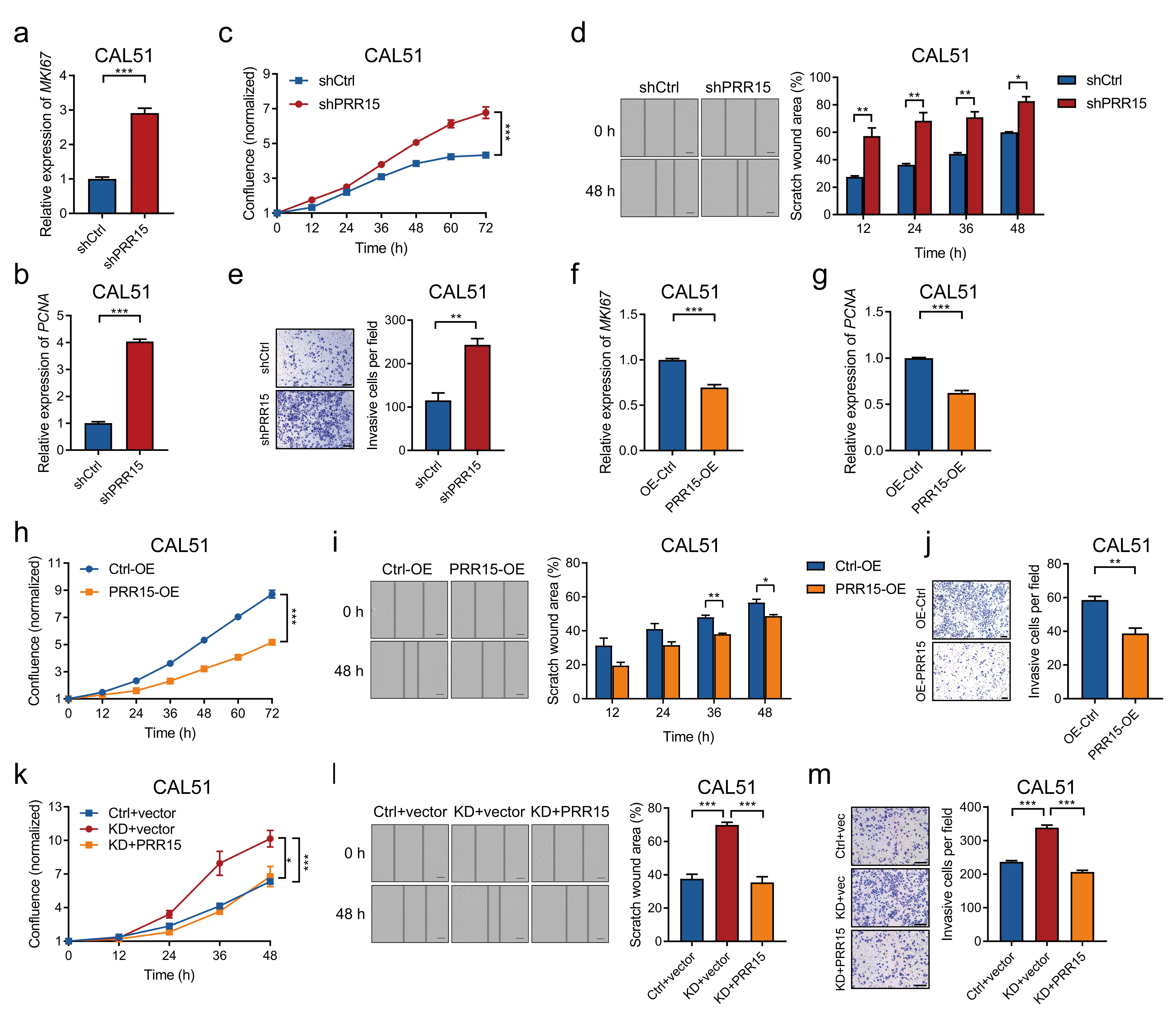

Supplement: Supplementary file 6 — Figure S5 [file 41419_2023_5746_MOESM6_ESM.tif]

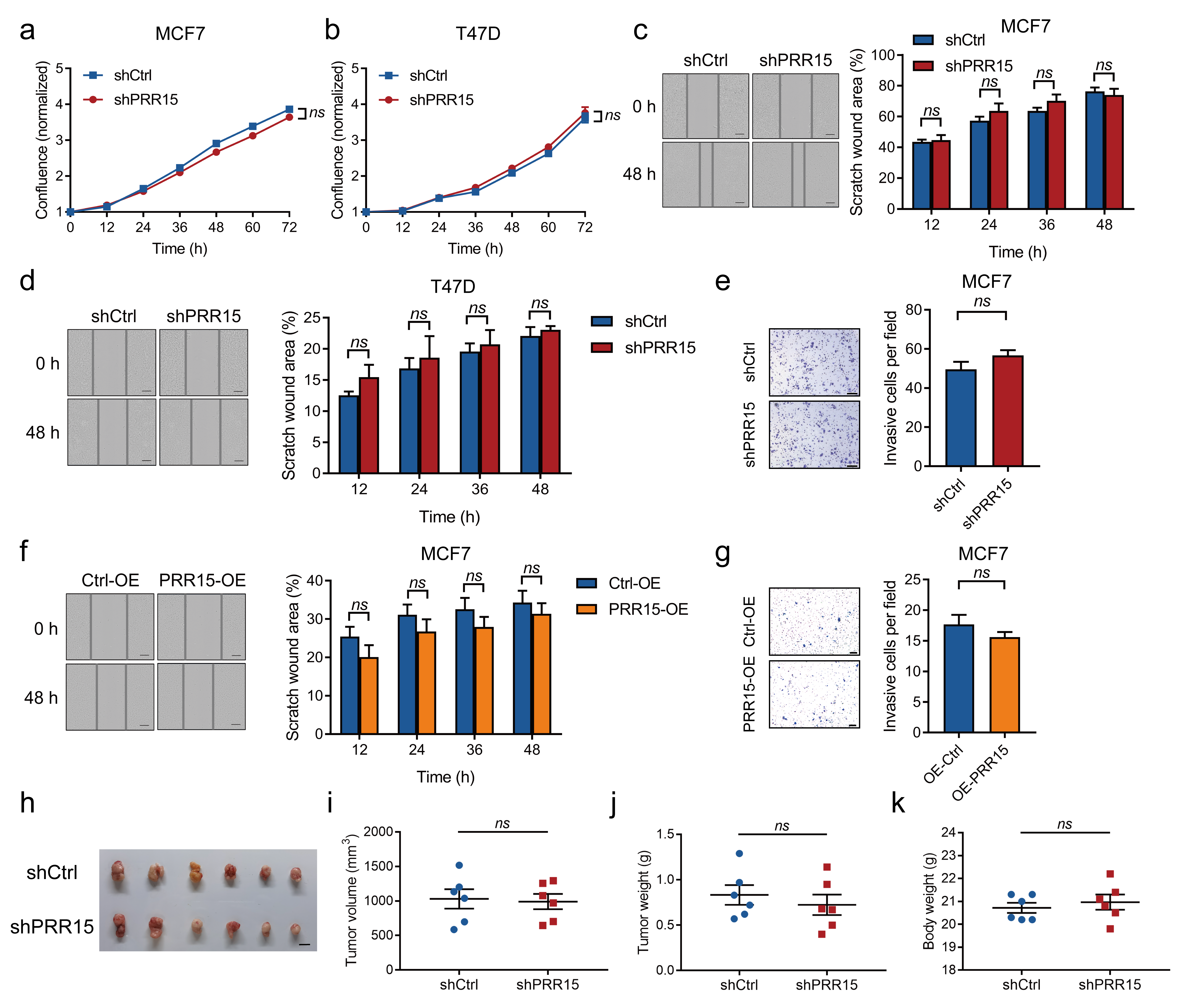

Supplement: Supplementary file 7 — Figure S6 [file 41419_2023_5746_MOESM7_ESM.tif]

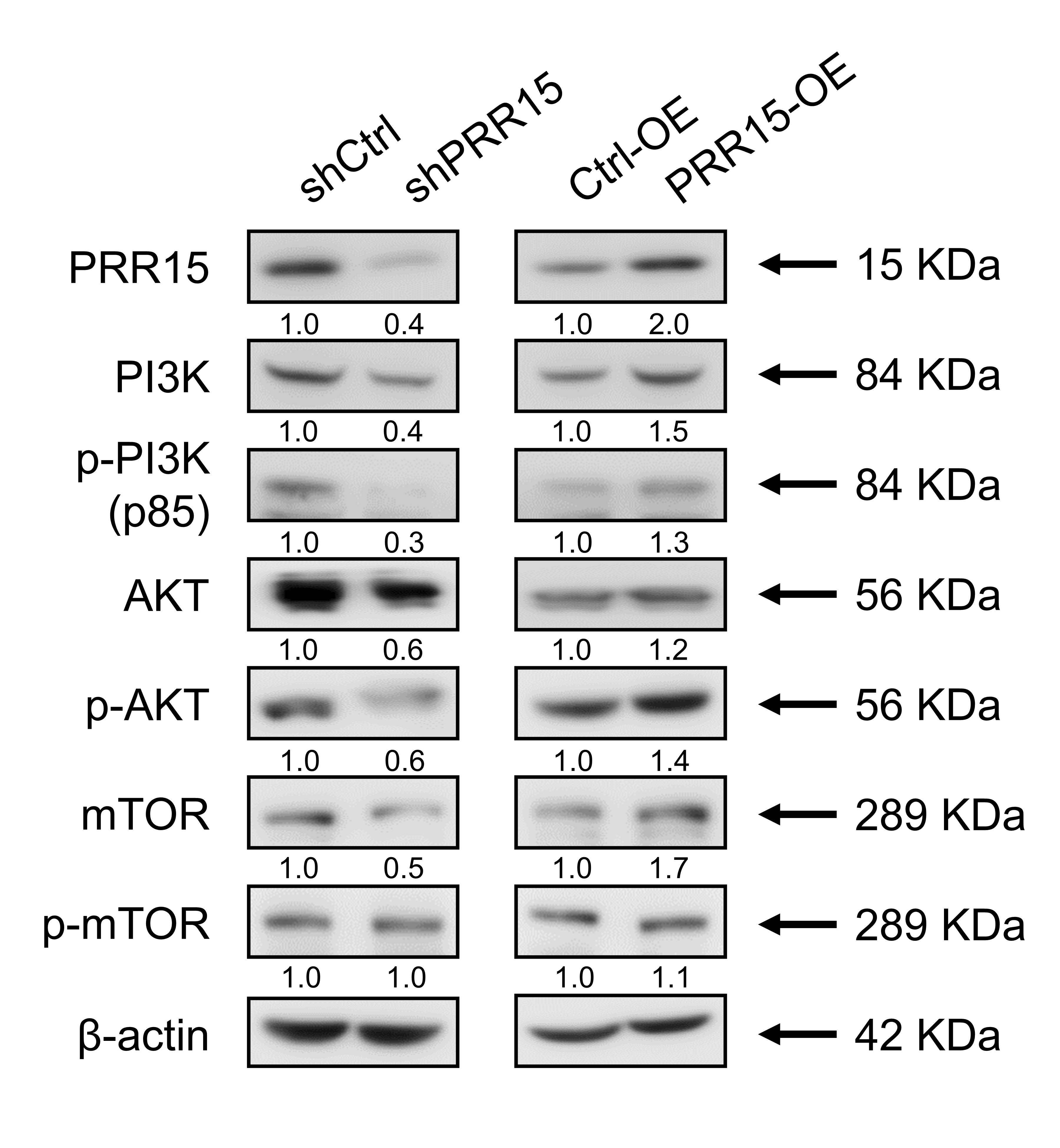

Supplement: Supplementary file 8 — Figure S7 [file 41419_2023_5746_MOESM8_ESM.tif]

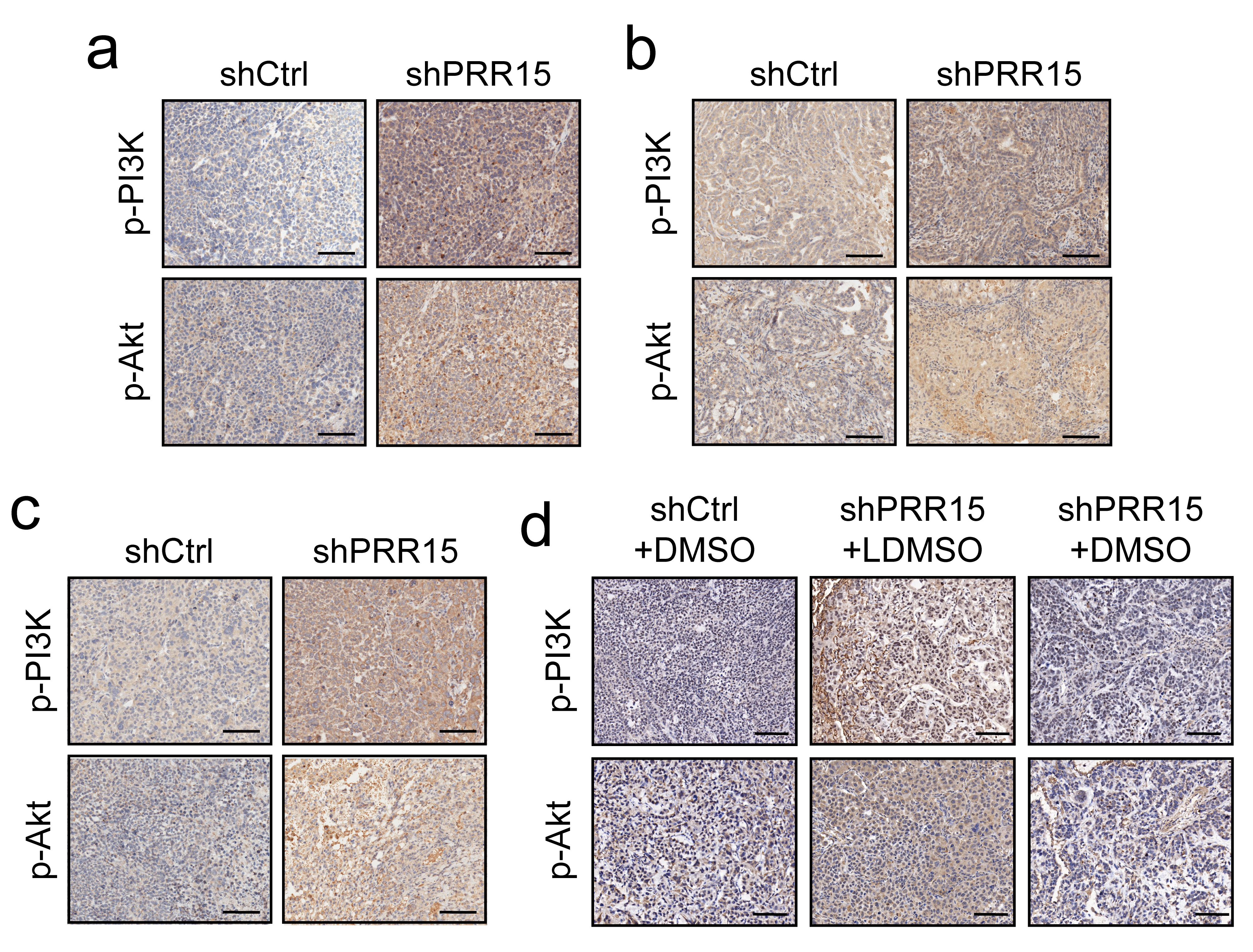

Supplement: Supplementary file 9 — Figure S8 [file 41419_2023_5746_MOESM9_ESM.tif]

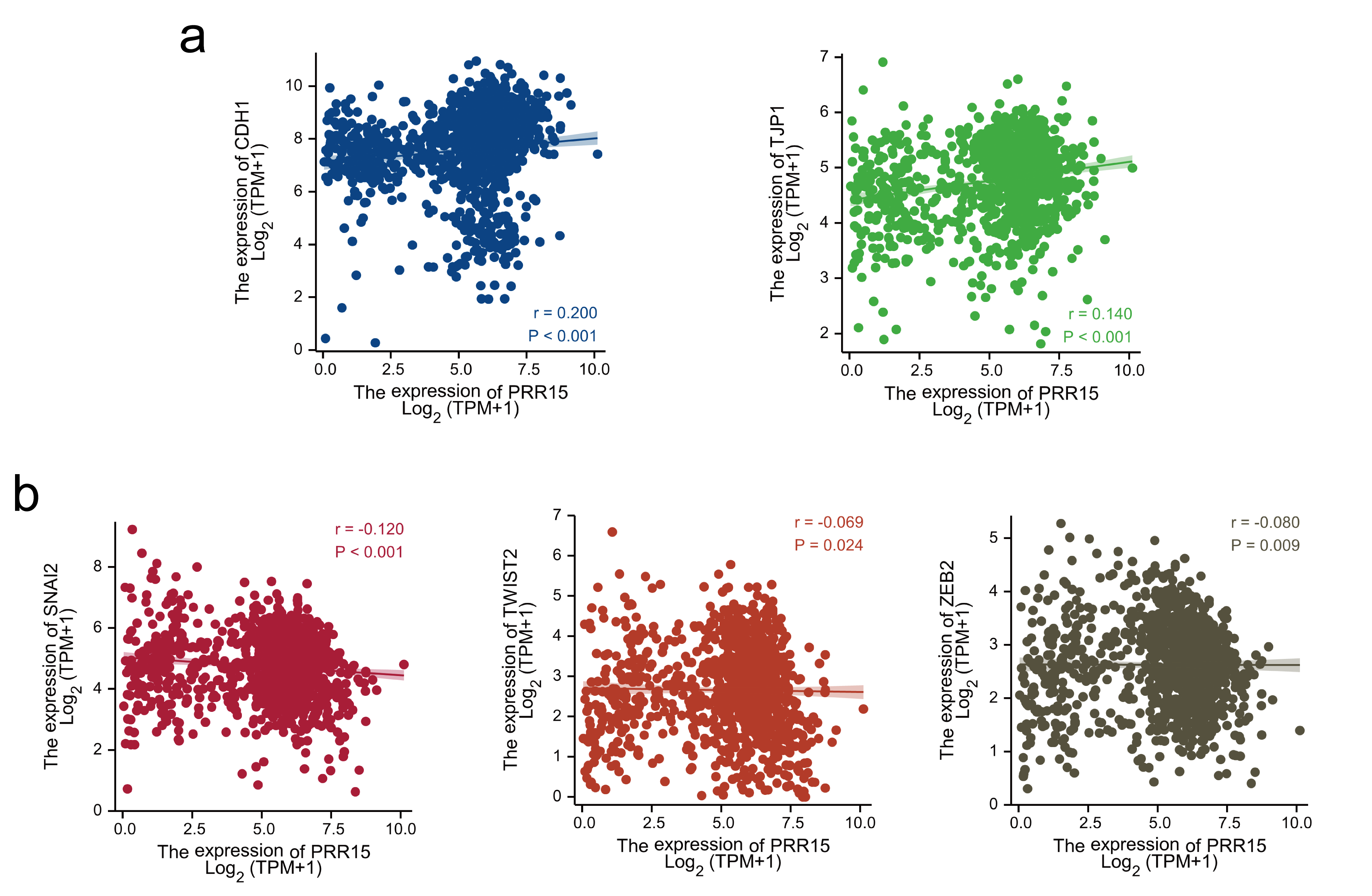

Supplement: Supplementary file 10 — Figure S9 [file 41419_2023_5746_MOESM10_ESM.tif]

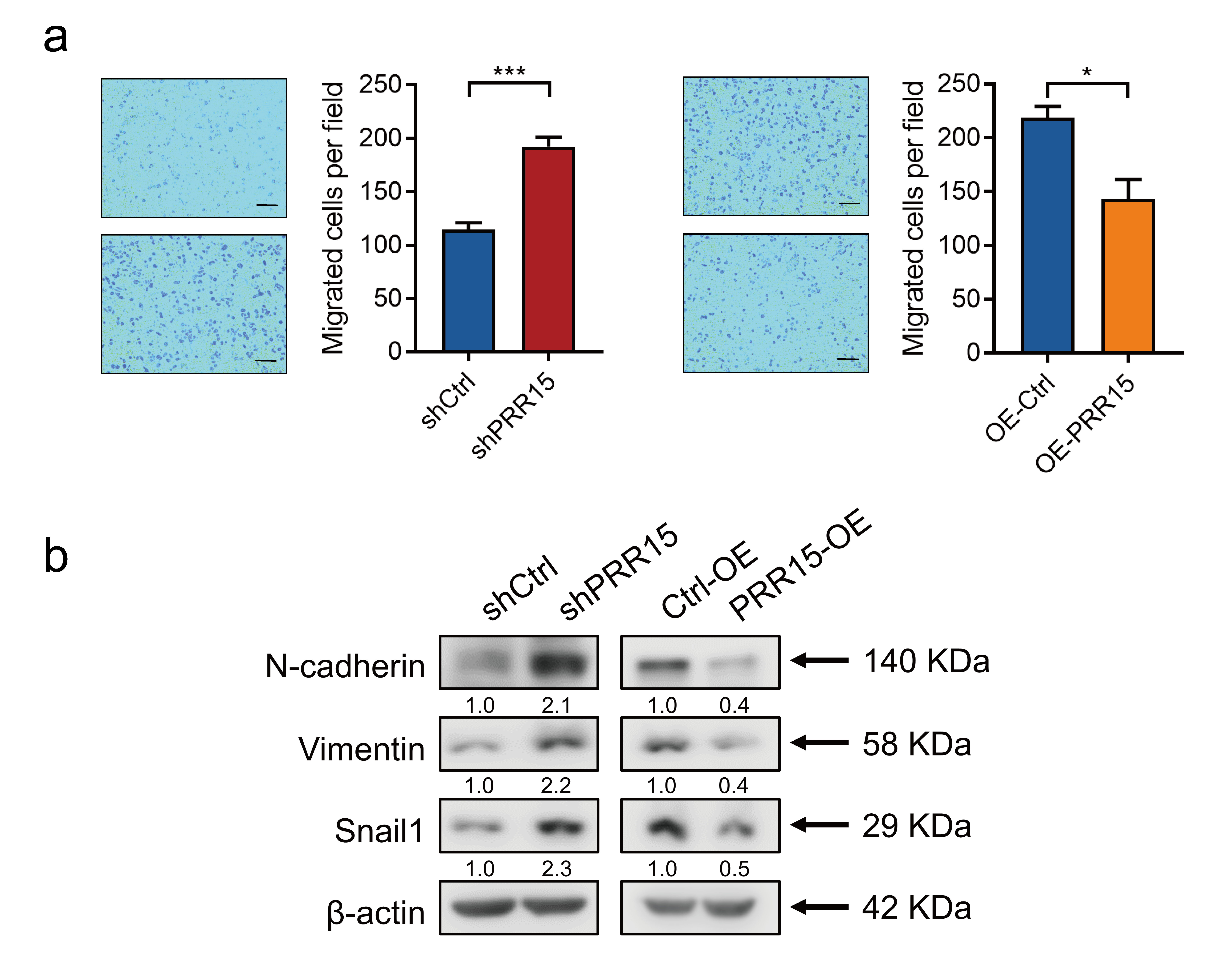

Supplement: Supplementary file 11 — Figure S10 [file 41419_2023_5746_MOESM11_ESM.tif]

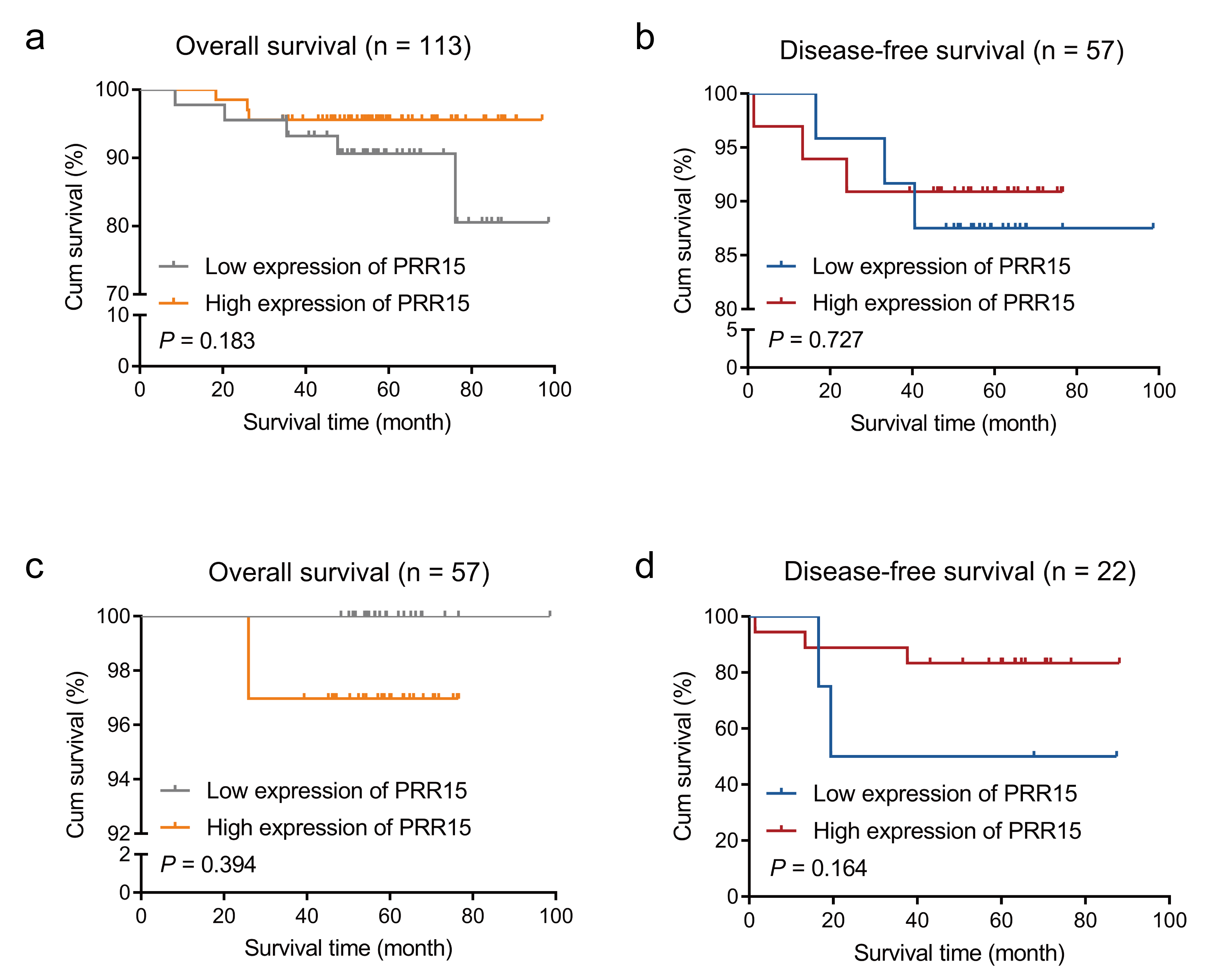

Supplement: Supplementary file 12 — Figure S11 [file 41419_2023_5746_MOESM12_ESM.tif]
