# Supplementary material for: PRR15 deficiency facilitates malignant progression by mediating PI3K/Akt signaling and predicts clinical prognosis in triple-negative rather than non-triple-negative breast cancer
Source: Cell Death Dis. 2023 Apr 18;14(4):272. doi: 10.1038/s41419-023-05746-8 (PMC10113191; doi:10.1038/s41419-023-05746-8)

Figure 4c

PRR15

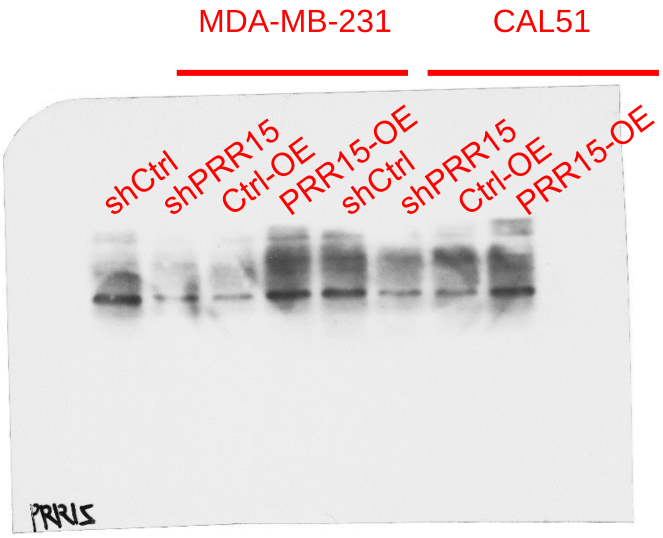

β-actin

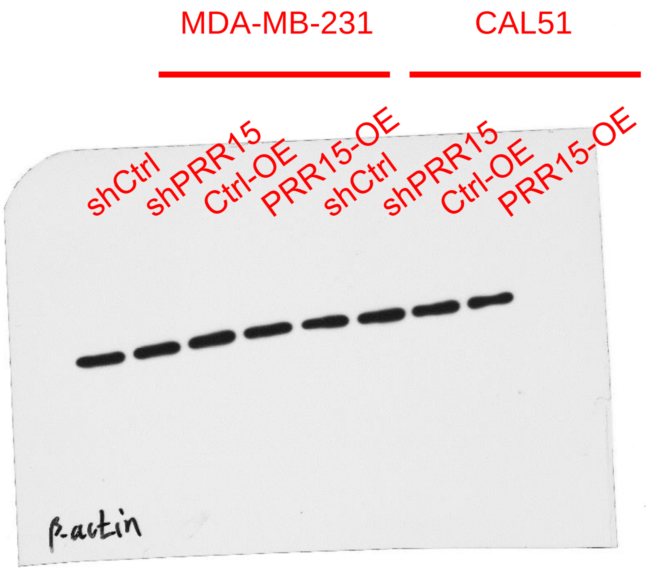

Figure 4c

MDA-MB-231
PI3K

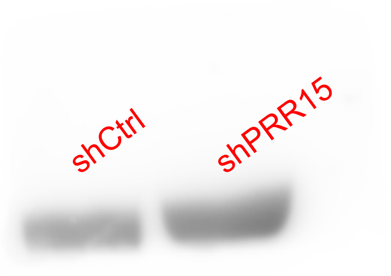

Figure 4c

MDA-MB-231
p-PI3K

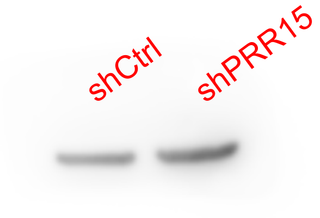

Figure 4c

MDA-MB-231
AKT

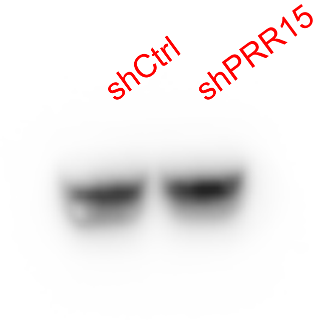

Figure 4c

MDA-MB-231
p-AKT

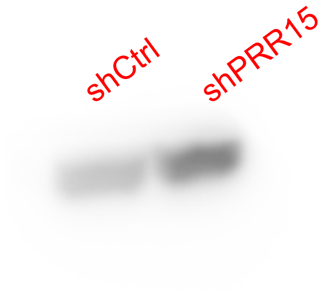

Figure 4c

MDA-MB-231
mTOR

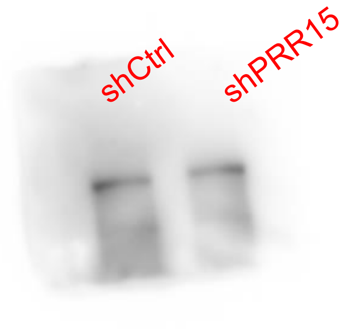

Figure 4c

MDA-MB-231
p-mTOR

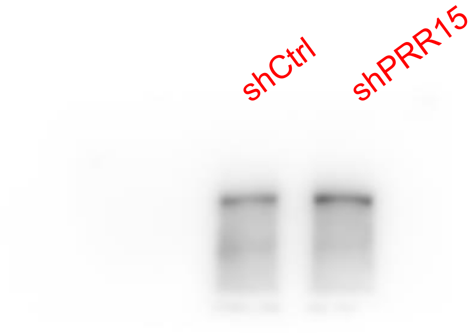

Figure 4c

CAL51
PI3K

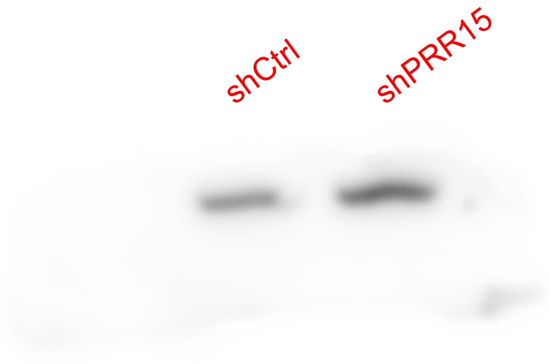

Figure 4c

CAL51
p-PI3K

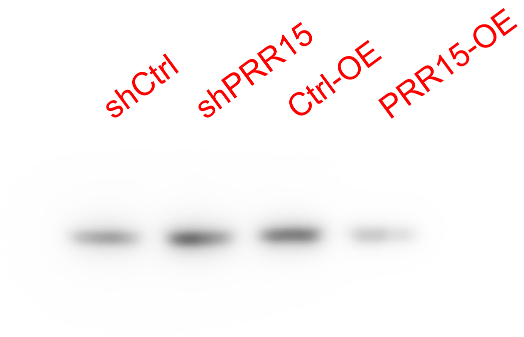

Figure 4c

CAL51
AKT

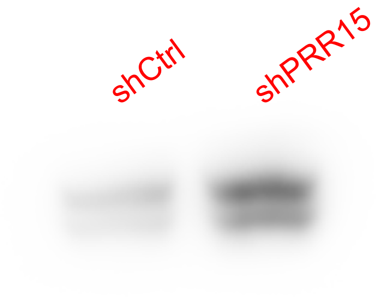

Figure 4c

CAL51
p-AKT

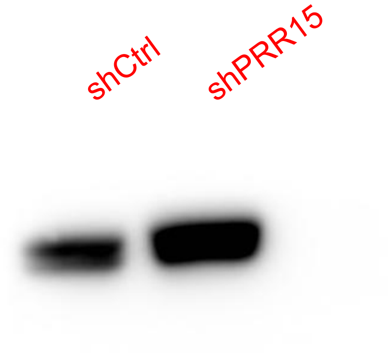

Figure 4c

CAL51
mTOR

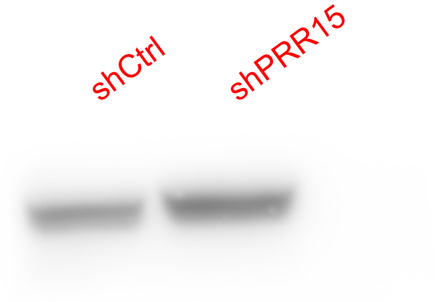

Figure 4c

CAL51
p-mTOR

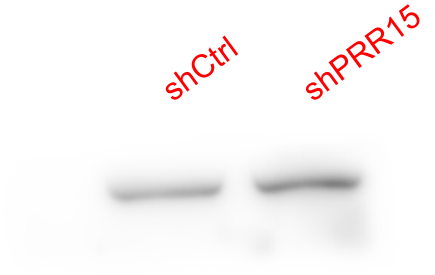

Figure 4c

MDA-MB-231
PI3K

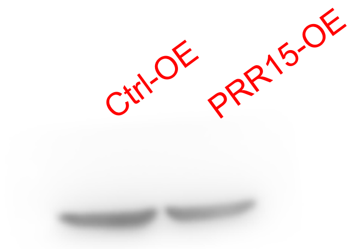

Figure 4c

MDA-MB-231
p-PI3K

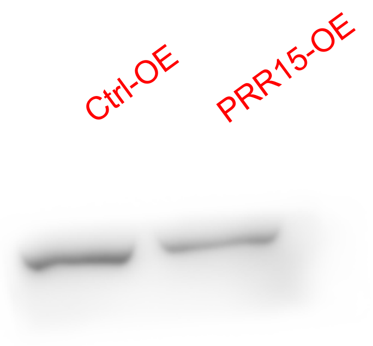

Figure 4c

MDA-MB-231
AKT

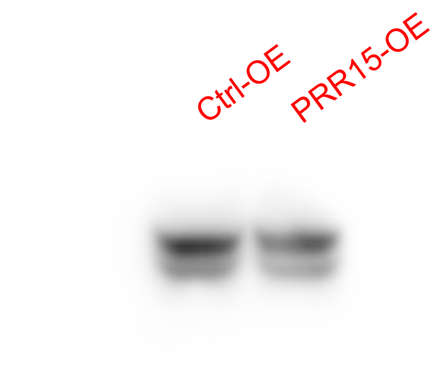

Figure 4c

MDA-MB-231
p-AKT

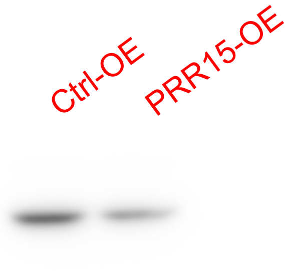

Figure 4c

MDA-MB-231
mTOR

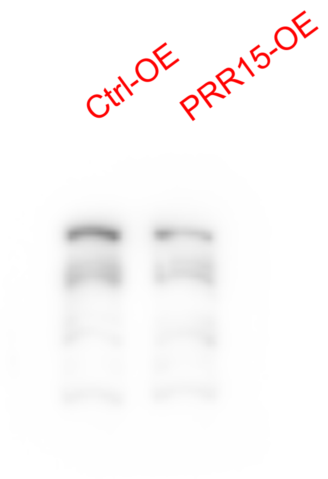

Figure 4c

MDA-MB-231
p-mTOR

Ctrl-OE PRR15-OE

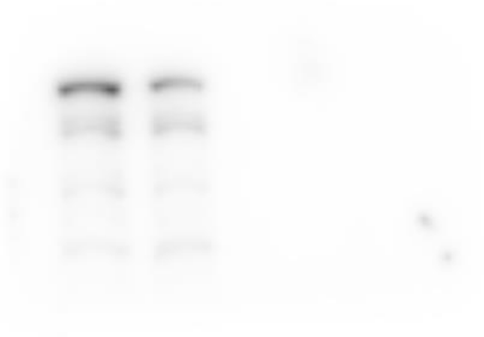

Figure 4c

CAL51
PI3K

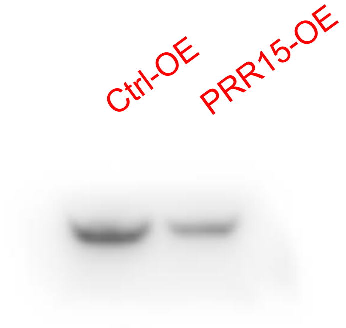

Figure 4c

CAL51
AKT

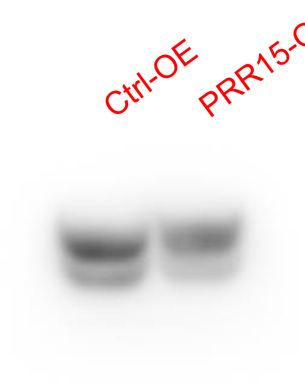

Figure 4c

CAL51
p-AKT

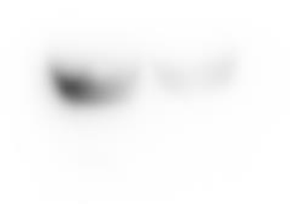

Figure 4c

CAL51
mTOR

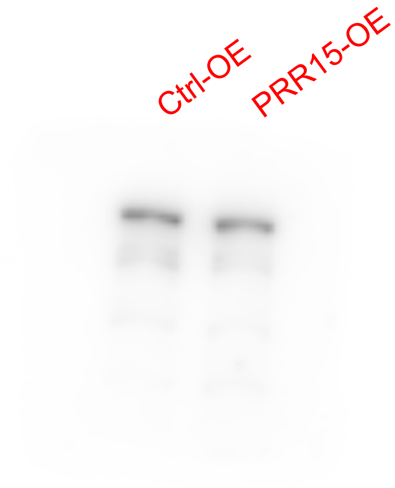

Figure 4c

CAL51
p-mTOR

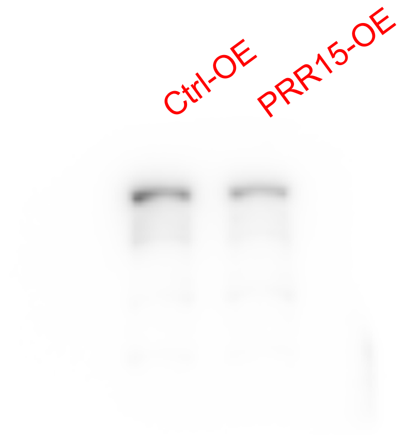

Figure 4p

MDA-MB-231
N-cadherin

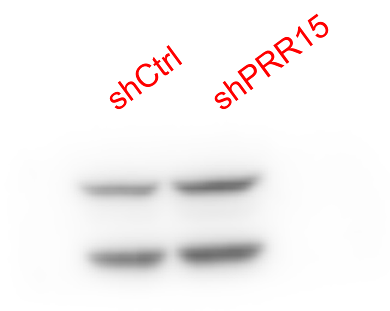

Figure 4p

MDA-MB-231
vimentin

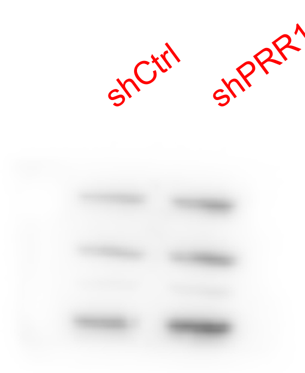

Figure 4p

MDA-MB-231
Snail1

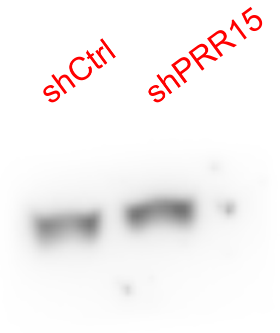

Figure 4p

MDA-MB-231
β-actin

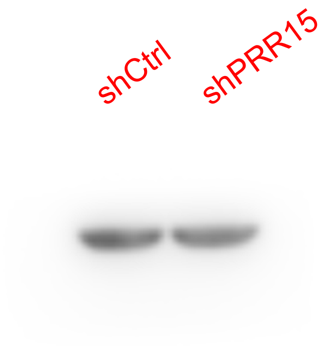

Figure 4p

MDA-MB-231
N-cadherin

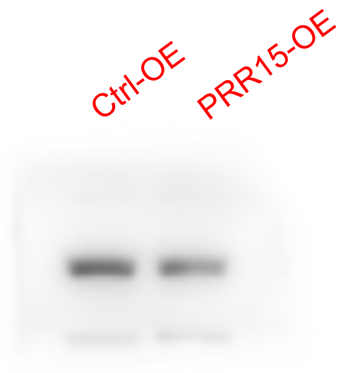

Figure 4p

MDA-MB-231
vimentin

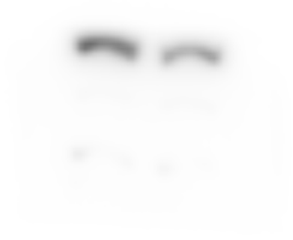

Figure 4p

MDA-MB-231
Snail1

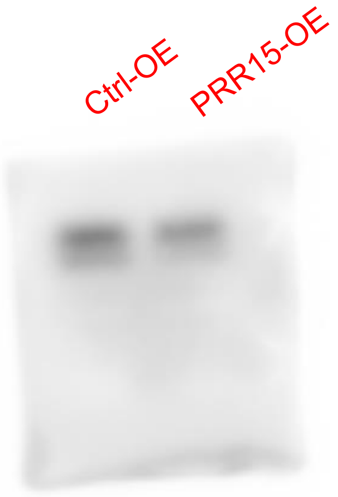

Figure 4p

MDA-MB-231
β-actin

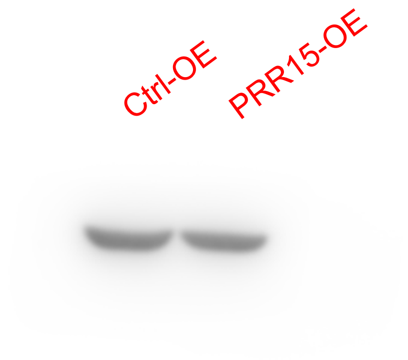

Figure S7

MCF7
PRR15

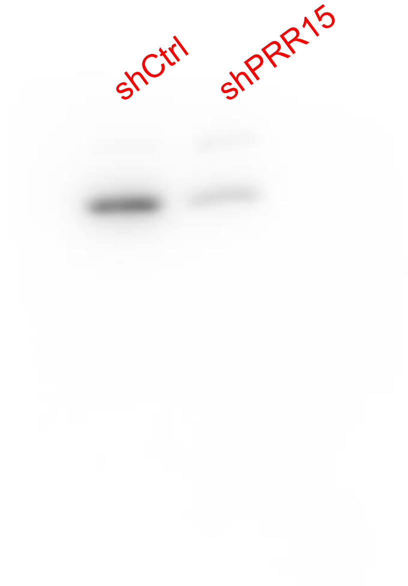

Figure S7

MCF7
PI3K

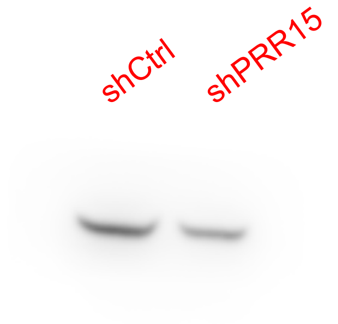

Figure S7

MCF7
p-PI3K

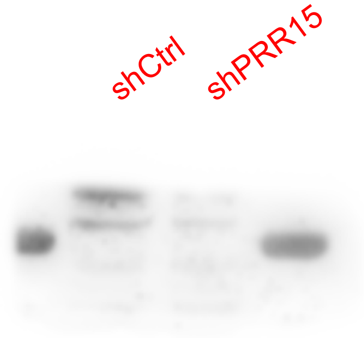

Figure S7

MCF7
AKT

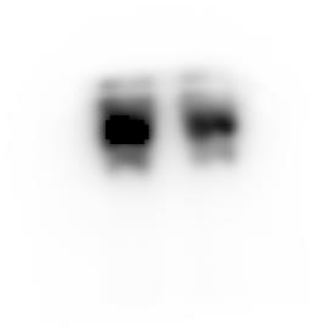

Figure S7

MCF7
p-AKT

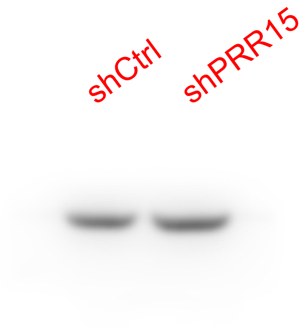

Figure S7

MCF7
mTOR

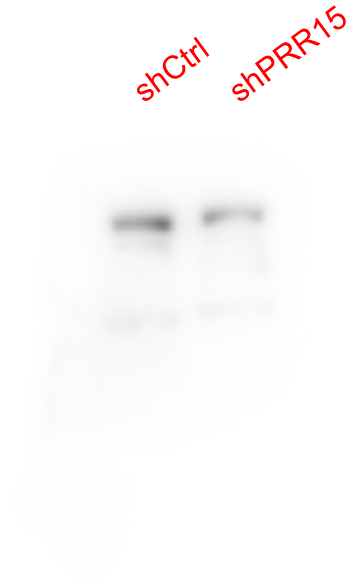

Figure S7

MCF7
p-mTOR

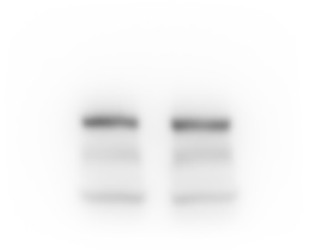

Figure S7

MCF7
β-actin

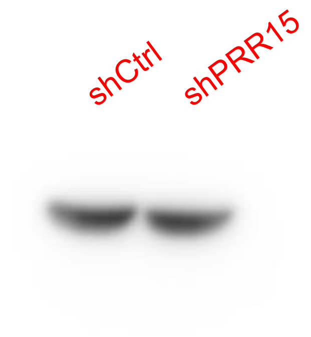

Figure S7

MCF7
PRR15

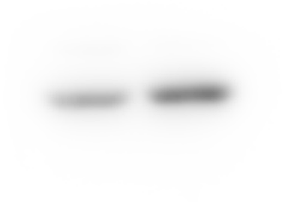

Figure S7

MCF7
PI3K

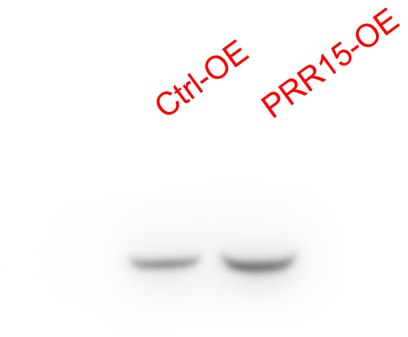

Figure S7

MCF7
p-PI3K

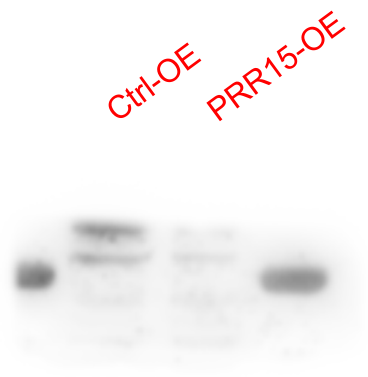

Figure S7

MCF7
AKT

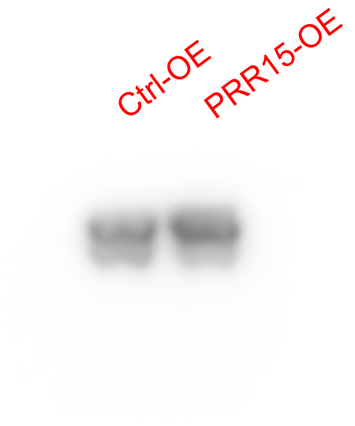

Figure S7

MCF7
p-AKT

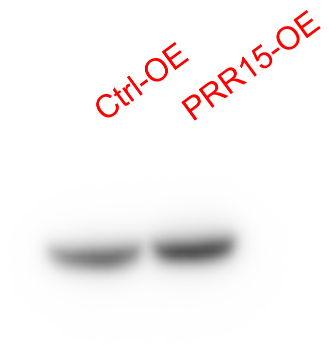

Figure S7

MCF7
mTOR

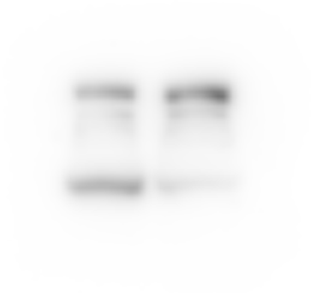

Figure S7

MCF7
p-mTOR

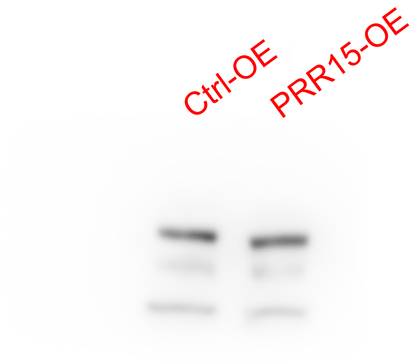

Figure S7

MCF7
β-actin

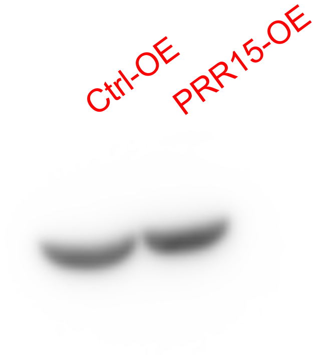

Figure S10b

CAL51
N-cadherin

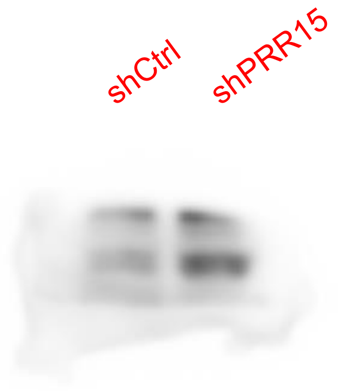

Figure S10b

CAL51
vimentin

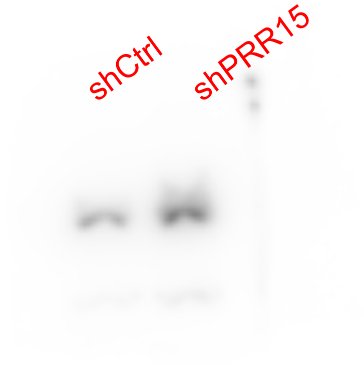

Figure S10b

CAL51
Snail1

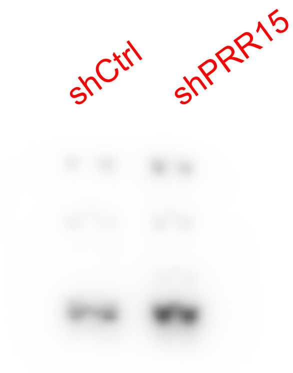

Figure S10b

CAL51
β-actin

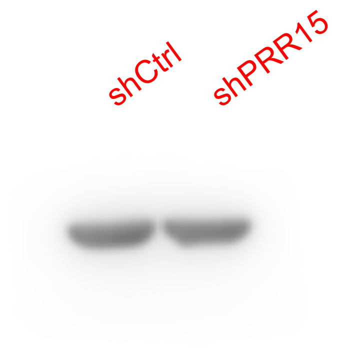

Figure S10b

CAL51
N-cadherin

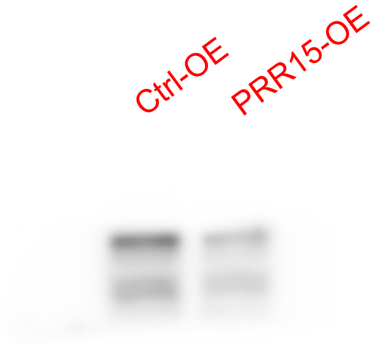

Figure S10b

CAL51
vimentin

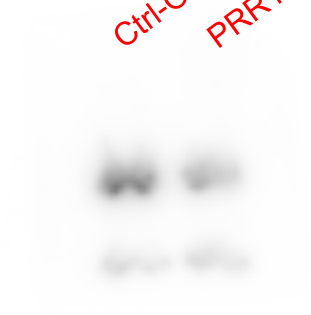

Figure S10b

CAL51
Snail1

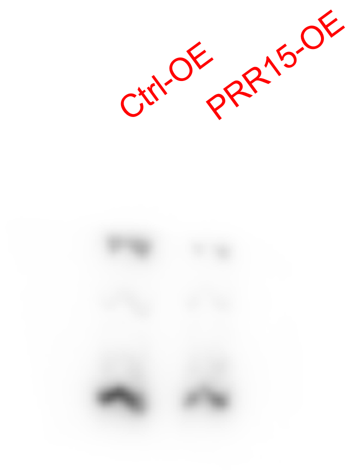

Figure S10b

CAL51
β-actin

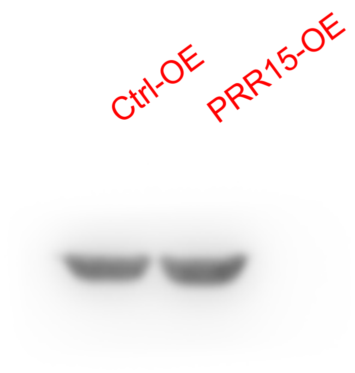

Supplement: Supplementary file 13 — Supplemental Material-WB (revised) [file 41419_2023_5746_MOESM13_ESM.pdf]
